# Supplementary material for: Basal complex: a smart wing component for automatic shape morphing
Source: Commun Biol. 2023 Aug 17;6:853. doi: 10.1038/s42003-023-05206-1 (PMC10435446; doi:10.1038/s42003-023-05206-1)
Supplement: Supplementary file 2 — Supplementary Information [file 42003_2023_5206_MOESM2_ESM.pdf]

# Supplementary Materials

## Basal Complex: A Smart Wing Component for Automatic Shape Morphing

*Sepehr H. Eraghi [1,2], Arman Toofani [1,2], Ramin J. A. Guilani [1,2], Shayan Ramezanpour [1,2], Nienke N. Bijma [3], Alireza Sedaghat [4], Armin Yasamandaryaei [3,4], Stanislav Gorb [3], and Hamed Rajabi [1,5]*

[1] Mechanical Intelligence (MI) Research Group, South Bank Applied BioEngineering Research (SABER), School of Engineering, London South Bank University, London, UK

[2] Faculty of Mechanical Engineering, University of Guilan, Rasht, Iran

[3] Functional Morphology and Biomechanics, Institute of Zoology, Kiel University, Kiel, Germany

[4] Department of Mechanical Engineering, Lahijan Branch, Islamic Azad University, Lahijan, Iran

[5] Division of Mechanical Engineering and Design, School of Engineering, London South Bank University, London, UK

## Supplementary Note 1

### Morphological investigation

### Damselfly *Ischnura elegans*

Scanning electron microscopy and micro computed tomography results revealed the presence of four different joint types in the basal complex of the forewing of *I. elegans*: rigidly fused joints (**Fig S3Di, Ei**), fused joints (**Fig S3Ei**), flexible joints (**Fig S3Di, Ei**) and bridge joints (**Fig S3Bi, Ci).** Since the fore and hindwings in damselflies exhibit a similar morphology, only the forewing of *I. elegans* is examined. According to SEM images, a dorsal-ventral asymmetry can be found in the shape of the joints in the arculus (**Fig S3Di, Ei**) and discoidal cell (**Fig S3Bi, Ci**), both parts of the basal complex of the wing. The most distal joint of the discoidal cell (**Fig S3Bi, Ci**) consists of a bridge joint built by MP and CuA. The arculus has two double flexible joints with RA and MP and two flexible-fused joint combinations at the intersections with RP and MA (**Fig S3Di, Ei**). Underneath RA and proximal to the arculus, a second vein called median vein runs parallel to RA (**Fig S3Di**). This vein is only visible on the dorsal side and is loosely connected to RA.

### Damselfly *Calopteryx splendens*

The results of morphology investigation of the wings of the damselfly *C. splendens* reveal differences between the joint types present on the dorsal and the ventral side of the arculus and the mediocubital bar (**Fig S5A, Bi, Ci, Di, Ei**). However, we did not observe any notable differences neither between sexes nor between the forewing and hindwing (**Fig S7**). The arculus is connected to the adjacent longitudinal veins via one double-fused joint with MA, one double-flexible joint with RA and two flexible-fused joints with RP and MP (**Fig S5Di, Ei**). MP has the flexible joint on the ventral side (**Fig S5Ei**), whereas RP has the flexible joint on the dorsal side (**Fig S5Di**). RP seems to be fused with the membrane close to the arculus. Furthermore, underneath RA and proximal to the arculus, a second vein called median vein runs parallel to RA (**Fig S5Di**). This vein is only visible at the dorsal side and is separated from RA by a narrow split. The mediocubital bar at the extreme distal part of the discoidal cell has intersections with three longitudinal veins: MA, MP and CuA (**Fig S5Bi, Ci**). MA is rigidly fused with the mediocubital bar on both sides, whereas CuA only shows a rigidly fused joint on the dorsal side. On the ventral side, however, it is attached to the mediocubital bar, which seems to be fused with the membrane posterior to MP. At the intercept with MP, the mediocubital bar has a bridge micro joint. On the dorsal side, Mb traverses MP. In addition, MP displays a change in morphology, and becomes slightly corrugated, exactly above the Mb on the ventral side (**Fig S5Ci**).

**Dragonfly *Sympetrum vulgatum***

The forewing and hindwing of the dragonfly *S. vulgatum* showed a different pattern of joint types and resilin patch distribution in the arculus and the discoidal cells than the two previously described damselfly species. Because fore- and hindwings are morphologically different, both wings are described separately. The arculus (**Fig 2Avii, viii, Fig S3v, vi**) and the three corners of the triangle, further referred to as vertex 1 (basal-anterior corner, **Fig 2Ai, Aii, Fig S3v, vi**), vertex 2 (apical-anterior corner, **Fig 2Ai, Aii, Fig S3Ai, Aii**) and vertex 3 (posterior corner, **Fig 2Aiii, Aiv, Fig S3Aiii, Aiv**), of the basal complex are examined in more detail.

**Forewing**

The arculus is connected to the adjacent longitudinal veins RA and MP with two double flexible joints and to RP with a flexible-fused joint, which exhibits a flexible hinge on the dorsal side. Underneath RA and proximal to the arculus, a second vein called median vein runs parallel to RA. This vein is only visible on the dorsal side (**Fig 2Avii**) and is not firmly connected to RA. The obtuse angle of vertex 1 (**Fig 2Avi, Avi**) is formed by the proximal and distal part of MP, Cr1 and Cr2. While Cr2 and the proximal part of MP are rigidly fused to each other on either side, the intercept with the distal part of MP is only rigidly fused on the ventral side and has a flexible joint on the dorsal side. Cr1 exhibits a double flexible joint combination. The acute angle of vertex 2 (**Fig 2Ai, Aii**) shows predominantly rigidly fused joints. Only Cr2 and Mb are flexibly joined on the dorsal and ventral side, respectively. However, at vertex 3 (**Fig 2Aiii, Aiv**) all four possible joint types are present: rigidly fused joints (between Mb and CuA dorsal), fused joints (between Mb and MP dorsal), flexible joints (between Mb and CuA ventral) and bridge joints (between MP and Mb ventral).

**Hindwing**

The arculus (**Fig S4Av, Avi**) exhibits an identical joint pattern in the hindwing as has been described for the forewing of *S. vulgatum*. They only differ in the connection of vertex 1 to MP of the arculus, with a double-flexible joint. Also, vertex 2 (**Fig S4Ai, Aii**) is broadly consistent with the joint pattern described for the forewing, except for having a double-flexible, instead of a single flexible joint with Cr2. However, vertex 3 (**Fig S4Aiii, Aiv**) at the tip of the triangle differs explicitly. It forms a five-rayed joint at the tip of the triangle. At the dorsal side Mb and the proximal and distal part of CuA are rigidly fused and traverse MP with a bridge joint. While on the ventral side all five longitudinal veins flatten before fusing with each other. The morphology at the fused part of the veins exhibits a more corrugated profile than on the longitudinal veins itself.

## Material composition investigation

### Damselfly *Ischnura elegans*

CLSM provided insights into the material composition and the distribution of resilin in the basal complex of *I. elegans*. Consistent with previous findings [1-3], the flexible protein resilin is predominantly found in form of patches that are associated with vein joints, creating flexible hinges. Besides, resilin is also found in the wing membrane.

Additionally, the presence of resilin in the joints shows a dorso-ventral anisotropy. Resilin patches on the ventral side are generally larger than those on the dorsal side (**Fig S3Bii, Cii, Dii, Eii**). On the dorsal side of the most distal joint of the discoidal cell, two relatively small resilin patches are located along the CuA (**Fig S3Bii**). On the ventral side, a large intersectional resilin patch running along the MP is present, with resilin patches along CuA.

On the dorsal side, the arculus is firmly attached to RA and MA (**Fig S3Dii**). Resilin is only found as a patch at the interval of MP and RP and, furthermore, as a barely visible lateral resilin patch along RP. However, on the ventral side, apparently more and larger resilin patches are present (**Fig S3Eii**). The anterior part of the arculus, limited to RA in one side and to MA on the other side, contain longitudinal resilin patches. Resilin patches are also present at the joints with RP and MP, although the latter contains only a small resilin patch. We did not observe any notable differences neither between sexes nor between the forewing and hindwing in *I. elegans* (**Fig S6**).

The observations made with the CLSM are consistent with those made with the wide-field fluorescence microscope (**Fig S3A**). To reveal a possible correlation between resilin patch distribution in the basal complex of the wing and the deformation of the wing during mechanical testing, we have visualized resilin containing joints by means of joint-by-joint mapping based on the WFM (**Fig S3A**). About half of all joints of the basal complex (n = 13) contain symmetrical joints with presence or absence of resilin on either side of the wing. The leading- and trailing edge of the wing contain joints, lacking any resilin, whereas joints along MP and RP exhibit double-sided resilin. The other half of the joints in the basal complex (n = 14) exhibit one sided resilin joints. Except for the joints along ScP and one joint on MP proximal to the arculus, all joints contain resilin only on the ventral side of the wing.

### Damselfly *Calopteryx splendens*

As for *I. elegans*, CLSM data show a dorsal-ventral asymmetry in resilin distribution in *C. splendens*. Only minor amounts of resilin could be found on the dorsal side of the basal complex (**Fig S5Bii, Cii, Dii, Eii**). On this side, neither the arculus nor the mediocubital bar is seen to have large resilin patches. Cross veins branching from the longitudinal veins (RP, MA, MP and CuA) close to the arculus and the mediocubital bar, show very small resilin gap patches. On the dorsal side, MP near to the arculus has small lateral resilin patches. The intercepts of the mediocubital bar and the three adjacent longitudinal

veins (MA, MP, CuA) are firmly connected, lacking any resilin. Furthermore, even the membrane at the intercept of MP and the mediocubital bar seem to be both sclerotised (**Fig S5Bii**).

However, on the ventral side of the basal complex, more resilin patches are present. Close to the arculus, longitudinal resilin patches run along RA, MA and the anterior part of the arculus between RA and RP. In addition, the intercept of the arculus with MP and RA contains resilin patches, which in the latter the resilin patch is very large. At the mediocubital bar, the three adjacent longitudinal veins (MA, MP and CuA) as well as the mediocubital bar itself contain resilin. MA and CuA have lateral resilin patches at intersect with the mediocubital bar, whereas the mediocubital bar itself contains lateral and longitudinal resilin patches. A longitudinal resilin patch is also found in MP, forming an intersectional resilin patch with mediocubital bar.

The observations made with the CLSM, are consistent with those made with the wide-field fluorescence microscope (**Fig S5A**). Joint-by-joint mapping revealed mainly symmetrical distribution of resilin patches (410 symmetrical joints and 78 one-sided resilin containing joints). Most of the resilin-containing joints can be found in the middle of the wing, far away from the leading and trailing edge. The three longitudinal veins of the leading edge spar, C&ScA, ScP as well as RA on the anterior side, lack any resilin, whereas IR, RP3, MA, MP and CuA show mainly double sided resilin joints. Some of those longitudinal veins (RA, RP3, MP, CuA, the composite vein and the trailing edge) contained additional one-sided resilin joints.

**Dragonfly *Sympetrum vulgatum***

In addition to the already observed dorso-ventral asymmetry in resilin patch distribution in *I. elegans* and *C. splendens*, the wings of *S. vulgatum* also show a dorso-ventral asymmetry in the magnitude of sclerotisation. Longitudinal veins are less sclerotised on the ventral side of the fore- and hindwing than on the dorsal side (**Fig 2B, Fig S3B**).

**Forewing**

The arculus shows relatively strongly sclerotised veins on the dorsal side. Only the joint between RA and the arculus exhibits a weaker sclerotisation. However, on the ventral side, except for MP, all veins exhibit weakly sclerotized cuticle and resilin is located as patches at the intercepts of RA and RP with the arculus and as lateral resilin patches at the anterior part of MA (**Fig 2Bviii**). On the dorsal side, only two resilin patches are present: a lateral resilin patch between the arculus and RP and a resilin gap patch between the arculus and MP (**Fig 2Bvii**). The obtuse angle of vertex 1 (**Fig 2Bv, Bvi**) is the only joint, containing a large resilin patch on the dorsal side and not on the ventral side. In addition, the longitudinal vein on the ventral side is less sclerotised. Vertex 2 and vertex 3 of the triangle show strongly sclerotised longitudinal veins with no resilin on the dorsal side (**Fig 2Bi-Biv**). On the ventral side, however, an inverse pattern is observed. All intersections of longitudinal veins contain resilin patches

and longitudinal veins show less sclerotisation.

### Hindwing

The morphological analysis of the arculus and vertex 2 (**Fig S4Bi, Bii, Bv, Bvi**) in the hindwing showed that the resilin distribution as well as the sclerotization of the veins are broadly consistent with those described in the forewing. Vertex 2 lacks the resilin patch between MA and Mb on the ventral side and instead exhibits a lateral resilin patch at the intercept of both veins. On the dorsal side of vertex 2, a patch between Mb and Cr2 is present. The only difference in the arculus of the fore and hindwings is the presence of the vertex 1 attached to MP via a resilin patch. A remarkably large resilin patch was found at the ventral side of the five-rayed joint at tip of the triangle (**Fig S4Biii, Biv**). Furthermore, lateral resilin patches along the longitudinal veins were present. However, on the dorsal side, only MP exhibits resilin patches attached to the fused intersection of the other three longitudinal veins.

The observations made with the CLSM, are consistent with those made with the wide-field fluorescence microscope (**Fig 2Bix, Fig S4Bvii**). In the fore- and hindwing of *S. vulgatum*, about half of all joints (42% and 51%, respectively) are symmetrical joints, i.e., either both sides contain or lack resilin. Joints lacking any resilin are mainly located at intercepts between adjacent cross veins. In addition, the longitudinal vein ScP and the trailing edge in the fore- and hindwing of *S. vulgatum* exhibit joints without resilin. However, the forewing exhibits more joints that lack resilin than the hindwing, whereas the hindwing shows more double-sided resilin-containing joints. The forewing only has double-sided resilin joints along the leading edge and MP, whereas the hindwing also exhibits double-sided resilin joint at MA and the anal loop of the wing. In both wings, the majority of one sided resilin joints contain the resilin patch on the ventral side of the wing; Joints exhibiting resilin only on the dorsal side are mainly located along RP and MP.

## Investigation of the mechanical behaviour

### Damselfly *Ischnura elegans*

To quantify the mechanical behaviour of the basal complex under loading, we measured the force required to deflect the forewing of *I. elegans*. Force-displacement curves show that force required to induce a displacement of about 10% of the lever arm (distance between the applied force and the fixation site) raises nonlinearly (**Fig S3H**). After reaching the maximum displacement, the force decreases due to the stress relaxation of the wing material. The mean bending moment required for the same displacement on the dorsal side is 21.60 ± 5.61 mg.mm ($N = 5$). This is comparatively smaller than the bending moment needed for the same displacement on the wing ventral side (28.50 ± 11.19 mg.mm ($N = 5$) (see supplement **Table S2** for force measurements). However, statistical analysis shows no significant difference in the bending moments between the dorsal and ventral sides (t-test, $t = -1.9583$,

df = 4, p = 0.1218).

Artificially shortened wings showed bending moment of 20.23 ± 11.06 mg.mm when subjected to loading on the dorsal side and 29.10 ± 14.90 mg.mm when subjected to loading on the ventral side. Comparing the forces needed to deflect the whole and the cut wing from the same side (**Fig S3I**), revealed no significant difference in bending moment (ANOVA, $F_{df=3,16}$ = 0.84, p = 0.4909).

Although the maximum bending moments revealed no significant difference during deformation from dorsal and ventral, a clear anisotropy can be seen in the deformation pattern of the intact wing when loaded dorsally and ventrally (**Fig S3L, P**). A force applied to the dorsal side causes the wing to rotate (**Fig S3L**). This is clearly visible by rotation of the trailing edge in the downward direction, while the leading edge stays almost in its initial position. Through the downwards bending of RA (**Fig S3L**), the dorsal-flexible joints, situated in the pleat valley of ScP, are stretched and thereby widen the angle, allowing the entire wing to rotate downwards. In addition, the double-flexible joint of RP is deflected downwards, increasing the observed downwards rotation of the wing (**Fig S10**). A rotation around MP, stretching the resilin located on the ventral side, slightly lifts the trailing edge upwards.

In contrast, a force applied to the ventral side results in camber formation of the wing (**Fig S3P**). The leading edge and the trailing edge move downwards, while the mid part of the wing moves in direction of the applied force. During camber formation in *I. elegans*, RP is elevated (**Fig S3P**). This upwards movement of RP causes RA to rotate along its axes. Resilin patches, situated at the joints, increase the chordwise flexibility of the wing and facilitate camber generation. During bending of longitudinal veins, the resilin patches get stretched. The resilin, located at the ventral side of the joint between RA and the arculus, is stretched, when the crease angle of RA is widened. The resilin, situated on the ventral side of the joint between RP and the arculus is also stretched, facilitating RP to move upwards. In addition to the observed rotation around RA, MA and CuA show an additional rotation along their axes, causing MP to raise and the trailing edge to depress (**Fig S10**). Most deformation occurs distal of the arculus. However, almost no difference in deformation pattern between the whole wing and the cut wing, deformed form the identical side, was visible. Although the pattern remains almost identical, the camber formation in cut wings is slightly shifted towards the wing base.

#### Damselfly *Calopteryx splendens*

Mechanical testing demonstrated that force, required to deflect the forewing of *C. splendens*, raises linearly by the applied displacement (**Fig S5F**). The mean maximum bending moment of 135.69 ± 28.79 mg.mm (N = 5) and 136.27 ± 48.09 mg.mm (N = 5) were applied during dorsal and ventral deflections, respectively (see supplementary **Table S2** for bending moments, measured for each deflection trail) (**Fig S5G**). Statistical analysis showed no significant difference between the bending moments required for the same magnitude of displacement on the dorsal and ventral sides (t-test, t = -0.0314, df = 4, p = 0.9764).

To characterise the contribution of the basal complex to the deformation of the wing, the more distal part of the wing has been removed. The rest part of the wing (basal complex) was deformed using the same method as that of the whole wing. Maximum bending moments of 99.97 ± 17.27 mg.mm (N = 5) on the dorsal side and 113.99 ± 63.73 mg.mm (N = 5) on the ventral side were measured for a displacement equal to 10% of the lever length (distance from the fixation to the location of the applied force) (**Fig S5G**). A comparison of the forces needed to deflect the whole and the cut wing from the same side, revealed no significant difference in bending moment (ANOVA, $F_{df=3,16}$ =0.8351, p = 0.4941). Similar to the observed dorsal-ventral symmetry of the bending moment, the deformation pattern shows hardly any difference during the deflection from the dorsal or the ventral side (Figure 10E,H). Force applied to both sides, results in a camber formation in the wing.

If the wing is deflected from the ventral side, the leading-edge spar (the first three longitudinal veins) is slightly tilted upwards (**Fig S10**) while the widening of the pleat angle of IR and MA, causes the trailing edge to rotate downwards, towards the side of the applied force. Especially distal to the medio-cubital bar, the downward rotation of the trailing edge is more intense. In contrast to the downwards rotation of the trailing edge, the central part of the wing is deflected in direction of the applied force. A pressure applied from the ventral side causes IR, RP3, MA and MP to move upwards, creating a cambered section. A pressure applied from the dorsal side will cause a downward bending of IR, RP3, MA and MP, creating a cambered section within the wing. The leading edge spar only slightly rotates downwards, mainly the widening of the pleat angle of RP2 enables camber generation. In addition, a slight rotation around RP3 and CuA (**Fig S10**) increases the steep angle of each valley, causing the trailing edge to raise, and thereby, reducing the camber. However, the tilt of the trailing edge distal to the medio-cubital bar is less clear on the ventral side (**Fig S10**) (quantitative force measurements are available in the supplementary **Table S2**).

*C. splendens* exhibits a symmetric deformation pattern under loads applied on dorsal and ventral surfaces. This means camber formation in both up- and downstroke, which is a characteristic for damselflies, using both half-strokes for aerodynamic lift generation [30] (although we found that *I. elegans* to do not follow this general trend) (**Fig S5**). The camber formation is highly dependent on the combination of joint types, resilin patches, and corrugations located on the basal complex of the wing (**Fig S5**). Double-sided resilin joints in damselflies may, therefore, reflect the symmetric loading in the upstroke and downstroke. However, there is no direct evidence that double-sided resilin joints contribute equally to dorsal and ventral flexibility [26]. Furthermore, the cross-section of corrugations in *C. splendens* wings shows a nearly symmetrical shape, which is mostly corrugated at the anterior and it is almost flattened at the posterior section, i.e., almost no pre-cambered on the ventral side (**Fig S5**). These corrugation patterns can further contribute to the symmetric deformation patterns of the wings during upward and downward deflections (see **Table S2**). Comparison between the deformation patterns of the whole wing and the cut wing, deformed form the same side, revealed hardly any

difference. Although the deformation pattern is the same, the camber formation in cut wings is shifted slightly towards the wing base.

**Dragonfly *Sympetrum vulgatum***

In the forewing of *S. vulgatum*, the mean bending moment required for the 10% displacement on the dorsal side was measured to be 211 ± 114 mg.mm (N = 5) (**Fig 2Cii**). This is larger than the bending moment measured on the ventral side 168 ± 52 mg.mm (N = 5) (**Fig 2Cii**) (see **Table S2** for force measurements). Statistical analysis showed no significant difference in the bending moment between the dorsal and ventral loadings (t-test, t = 1.147, df = 4, p = 0.3153).

The hindwing of *S. vulgatum* showed a relatively similar mean bending moment on the dorsal and ventral side. A bending moment of 134 ± 77 mg.mm (N = 5) was required to generate a 10% dorsal deflection, whereas a slightly lower bending moment of 119 ± 52 mg.mm (N = 5) was measured for the displacement on the ventral side (**Fig S4Cii**). Statistical analysis for the hindwing also revealed no significant difference between the bending moments required for a 10% displacement on the dorsal and ventral side (t-test, t = 0.7518, df = 4, p = 0.494) (quantitative force measurements are available in the **Table S2**).

Even though no significant differences were measured during deformation of the fore- and hindwing from the dorsal and ventral side, a clear asymmetry was observed in deformation patterns between the dorsal and ventral loadings in the forewing (**Fig 2Civ, Cvi, Cviii, Cx**) and hindwing (**Fig S4Civ, Cvi, Cviii, Cx**). Almost no difference in deformation pattern (deformed form the identical side) was visible between the whole wing and the cut wing for both fore- and hindwing. Here again, although the deformation pattern remains unchanged, the camber formation in cut wings was shifted slightly towards the base of the wing.

In *S. vulgatum*, cut wings showed bending moments during deflection of the forewing of 175 ± 88 mg.mm from the dorsal and 128 ± 36 mg.mm from the ventral side (**Fig 2Cii**). For the hindwing, the bending moment was 86 ± 37 mg.mm for the dorsal deflection and 82 ± 40 mg.mm for the ventral deflection (**Fig S4**). Comparing the forces needed to deflect the whole and the cut wing from the same side, revealed no significant difference in bending moment in the forewing (ANOVA, $F_{df=3,16}$ = 0.9221, p = 0.4526) and hindwing (ANOVA, $F_{df=3,16}$ = 1.1067, p = 0.3754) (**Fig 2Cii, Fig S4**)

Although the bending moment needed for 10% displacement was relatively smaller in hindwings than in forewings, no significant differences in bending moment between fore- and hindwing was detected during deflection from the dorsal side in intact and cut wings (ANOVA, $F_{df=3,8}$ = 0.8367, p = 0.5106) as well as between fore- and hindwing deformed from the ventral side in intact and cut wings (ANOVA, $F_{df=3,8}$ =1.1067, P=0.3754).

## Supplementary Figures & Tables

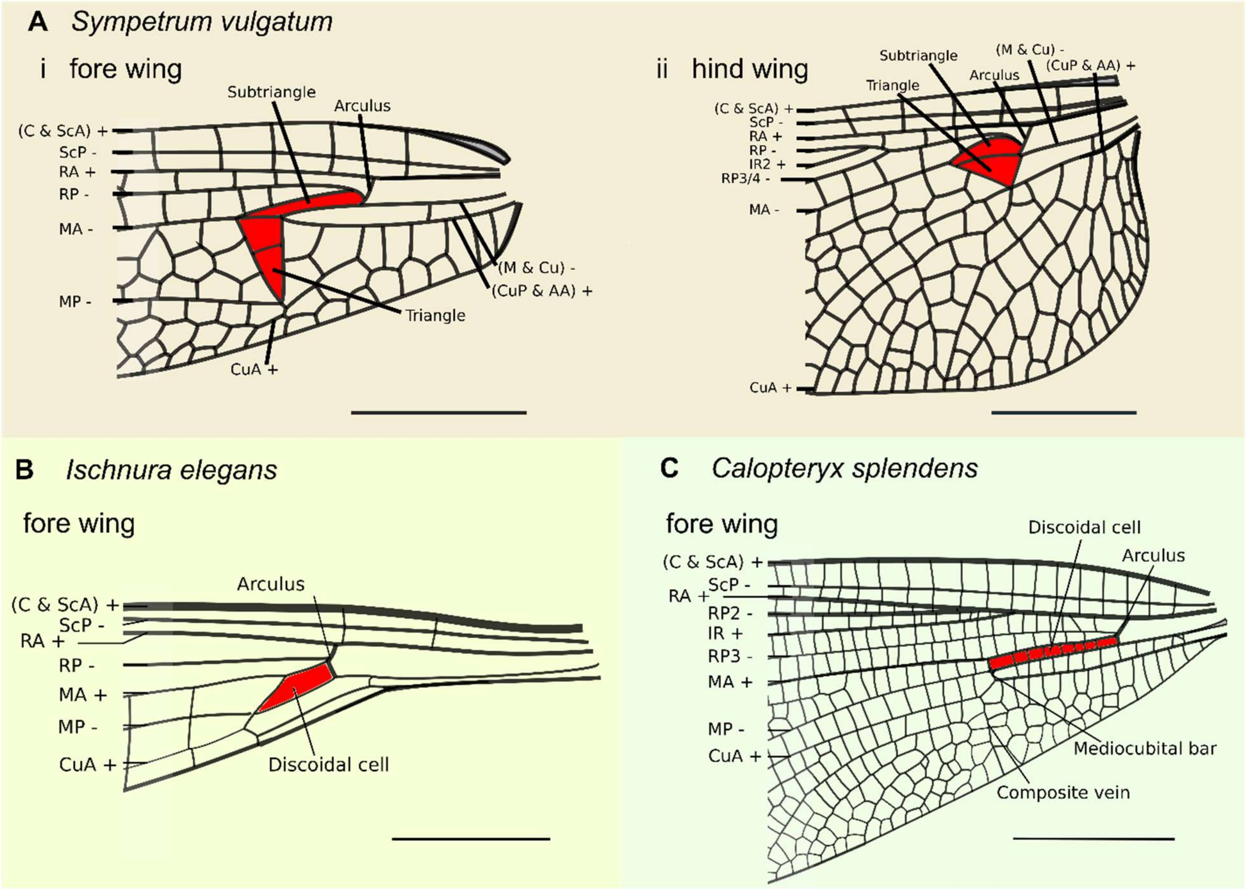


**Figure S1.** Basal complex of the dragonfly *S. vulgatum* forewing and hindwing (A, B), the damselfly *I. elegans* forewing (C) and the damselfly *C. splendens* forewing (D). Wings are shown from the dorsal side with "+" and "-" indicating if veins are raised (hill) or lowered (valley) in reference to the midline of the wing. Red areas show the discoidal cells. AA - anal vein anterior, C - costal vein, CuA -cubital vein anterior, CuP - cubital vein posterior, IR - intercalated vein, MA - median vein anterior, MP- median vein posterior, RA - radial vein anterior, RP -radial vein posterior, ScA - subcostal vein anterior, ScP - subcostal vein posterior. Scale bars = 0.5 cm (A, B, D), 0.2 cm (C).

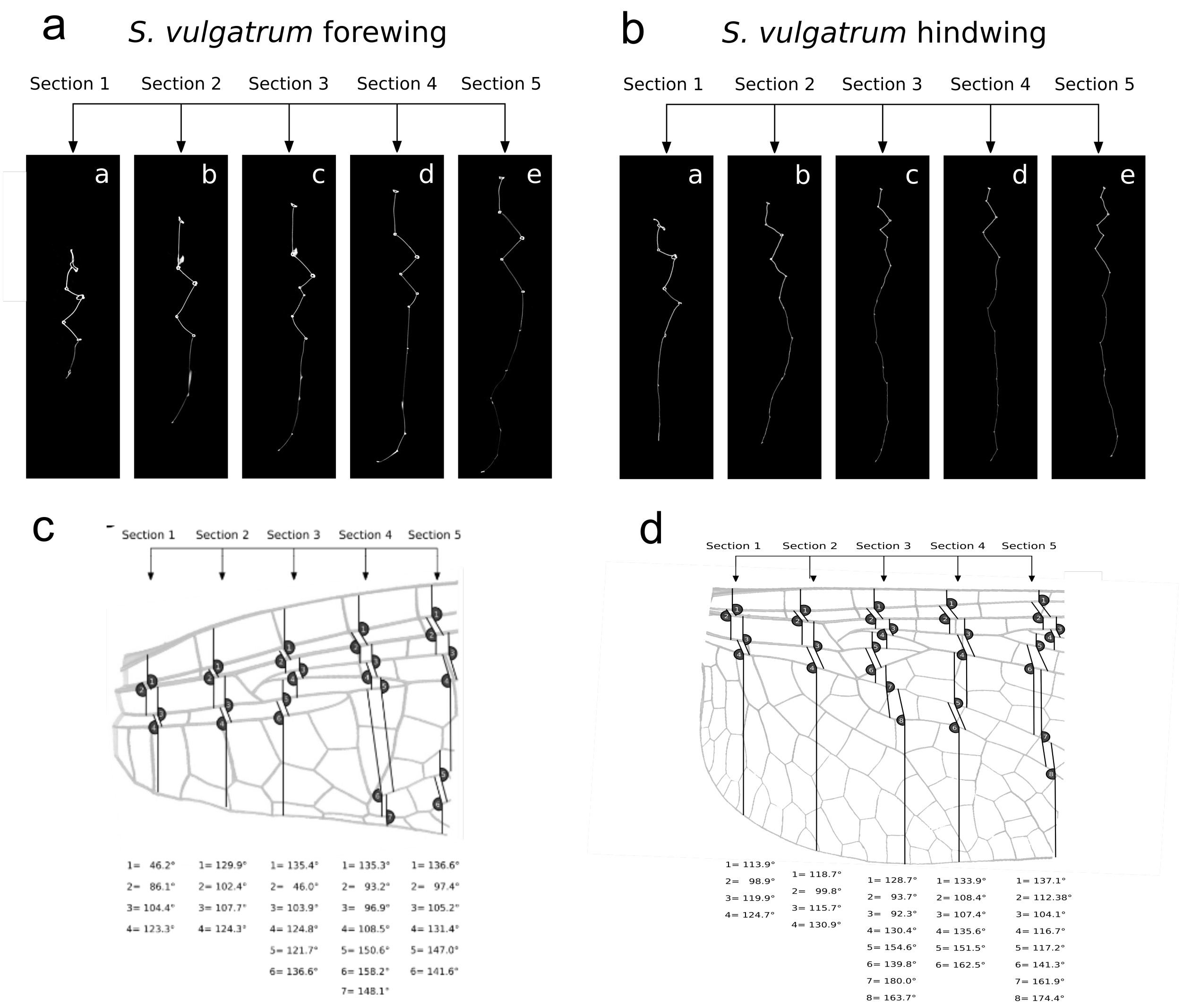


**Figure S2**. Cross sections through the basal complex of *S. vulgatum* (a,b). Reconstructed micro-CT images show the corrugation of the wing in five section. Section 1 is most basal, section 5 is at the end of the basal complex. All sections are at equal distance to each other. Contrast of micro-CT images is increased. Corrugation profile of S. *vulgatum* (c,d). Angles between neighbouring longitudinal veins are measured along five sections of the basal complex. Calculated angles are listed below each section.

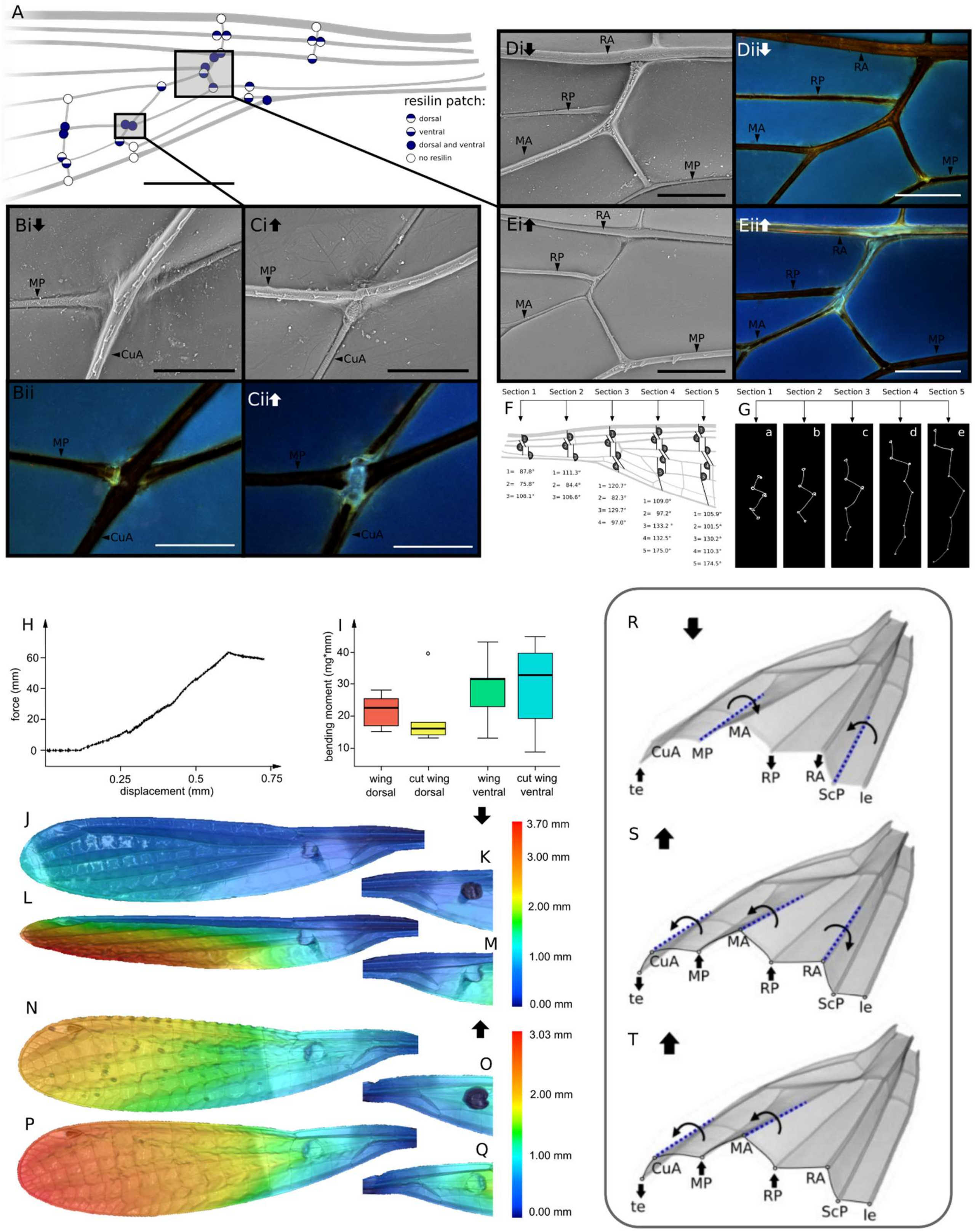


**Figure S3.** Structural, material, and mechanical characterization of the basal complex of damselfly *I. elegans*. Distribution map of resilin patches in the forewing of *I. elegans*, based on fluorescence microscopy (A). The occurrence of resilin is illustrated by blue colour according to its location in the wing (dorsal or/and ventral). White circles indicate firmly connected joints, lacking resilin (A). SEM images of the most distal part of the discoidal cell (Bi,Ci) and arculus (Di,Ei). Dorsal and ventral sides are indicated

by downward pointing and upward pointing arrows, respectively. Slight differences between dorsal and ventral side are due to the use of different specimen. Ac - arculus, CuA - cubital vein anterior, M - median vein, MA - median vein anterior, MP - median vein posterior, RA - radial vein anterior, RP - radial vein posterior. CLSM (maximum intensity projection), showing the occurrence of resilin in the arculus (Dii,Eii) and the most distal part of the discoidal cell (Bii,Cii). Blue colour indicates the presence of resilin, red colour show relatively strongly sclerotised cuticle, green colour show weakly sclerotised cuticle. Downward pointing and upward pointing arrows indicate the dorsal and ventral side of the wing. Slight differences between dorsal and ventral side are due to the use of different specimen. Mechanical testing of wing deformability in *I. elegans*. Intact and cut forewings, were deflected from dorsal and ventral. Representative force displacement curve for 10% deflection (H). Box-and-Whisker plots for resulting bending moments for the four experimental conditions (I). Resulting height profile before (J,K,N,O) and after (L,M,P,Q) deflection. Downward and upward pointing arrows indicate the dorsal (J-M) and ventral (N-Q) loadings, respectively. Corrugation profile of *I. elegans* (F). Angles between neighbouring longitudinal veins are measured along five sections of the basal complex (F). Calculated angles are listed below each section (F). Cross sections through the basal complex of *I. elegans* (G). Reconstructed micro-CT images show the corrugation of the wing in five section (G). Section 1 is most basal, section 5 is at the end of the basal complex. All sections are at equal distance to each other (G). Schematic drawing of the basal complex of *I. elegans*, showing the effect of dorsal (T) and ventral (R,S) applied forces on the longitudinal vein RP. Figures (S) and (T) exhibit the observed deformation in the basal complex of *I. elegans*. Figure (R) represents the suggested mechanism of camber formation according to Wootton and Newman (2008). The basal complex of the wing is shown from the dorsal side. Arrows indicate the movement of single longitudinal veins.

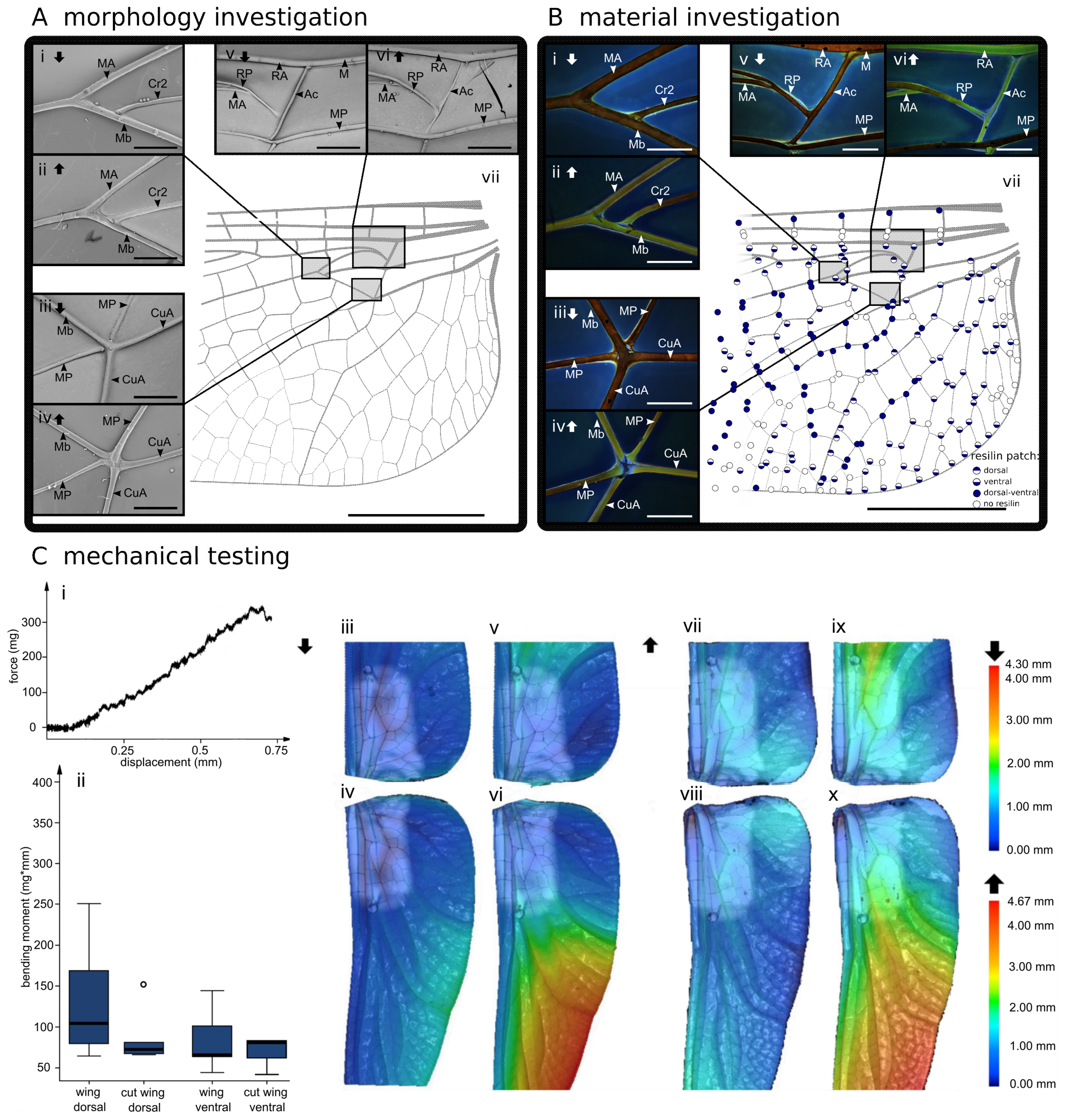


**Figure S4.** Structural, material, and mechanical characterization of the basal complex of the hindwing of the dragonfly *S. vulgatum*. SEM and CLSM images of the arculus (Av, Avi, Bv, Bvi) and apical-anterior corner (Ai, Aii, Bi, Bii) and the posterior corner (Aiii, Aiv, Biii, Biv). Dorsal and ventral sides are indicated by downward pointing and upward pointing arrows, respectively. Slight differences between the dorsal and ventral side are due to the use of different specimen. Ac - arculus, Cr - cross vein, Cu - cubital vein, CuA - cubital vein anterior, M - median vein, MA - median vein anterior, Mb - mediocubital bar, MP - median vein posterior, RA - radial vein anterior, RP - radial vein posterior. Distribution map of resilin patches in the hindwing of *S. vulgatum* based on fluorescence microscopy (Bvii). The occurrence of resilin is illustrated by blue colour according to its location in the wing (dorsal or/and ventral). White circles indicate firmly connected joints, lacking resilin. The gray area illustrates the anal loop. Image has

been modified from Appel and Gorb (2014) (Bvii). Mechanical testing of wing deformability in *S. vulgatum* (C). Intact and cut hindwings were deflected from dorsal and ventral sides. Representative force displacement curve for 10% deflection in the hindwing (Ci). Box-and-Whisker plots for resulting bending moments for the four experimental conditions in for hindwing (Cii). Measured height profiles of the hindwing (Ciii - Cx) of *S. vulgatum*. Intact and cut hindwings were deflected from dorsal and ventral. Resulting height profiles before (Ciii, Civ, Cvii, Cviii) and after (Cv, Cvi, Cix, Cx) 10% deflection. Downward and upward pointing arrows indicate the side from which deformation forces were applied (dorsal side Ciii-vi and ventral side Cvii-x, respectively). Scale bars: 0.5 mm (Avii,Bvii) for schematic drawings, 250 µm (Ai – Aiv, Bi - Biv), 500 µm (Av, Avi, Bv, Bvi).

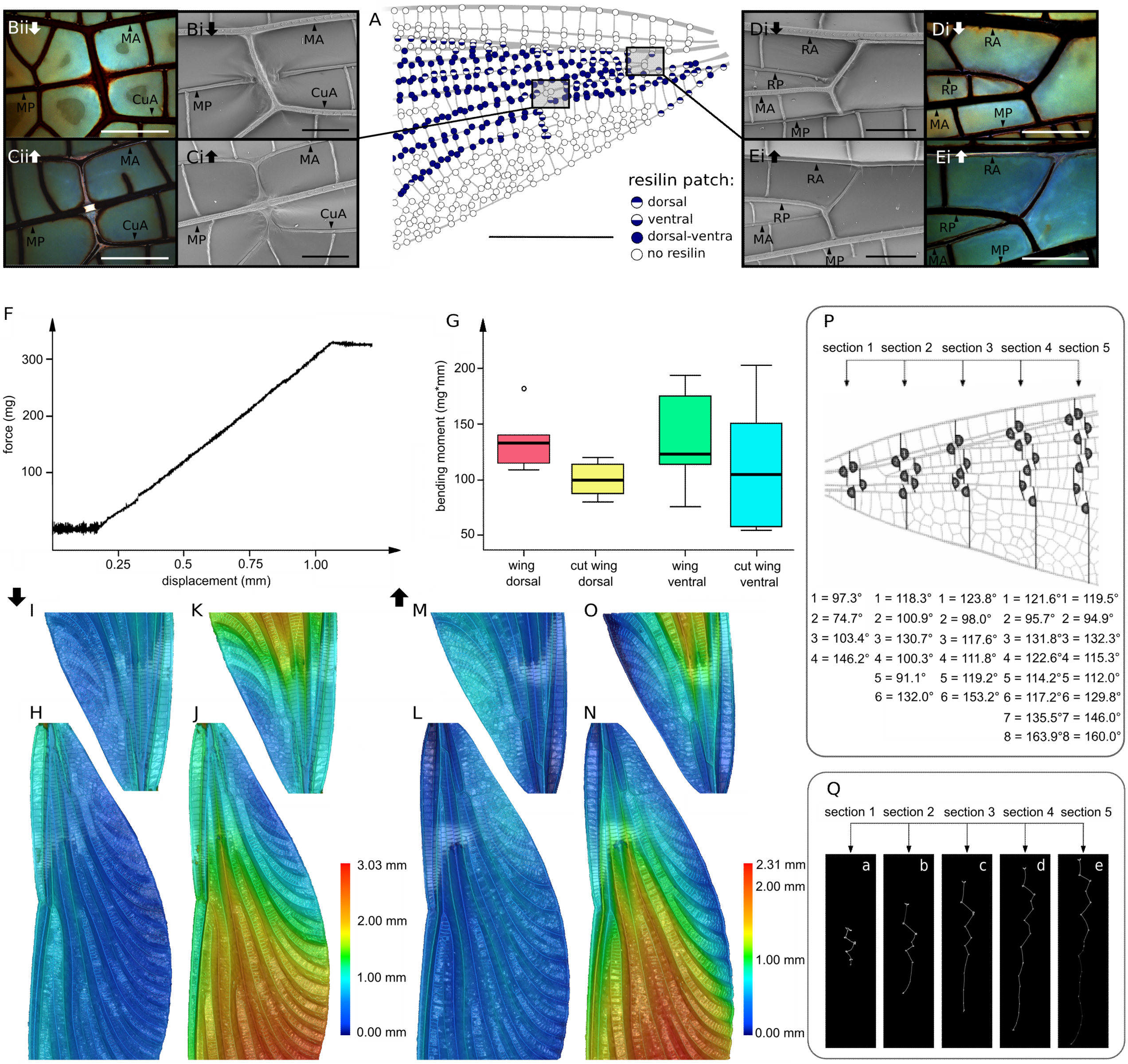


**Figure S5.** Structural, material, and mechanical characterization of the basal complex of damselfly *C. splendens*. Distribution map of resilin patches in the forewing of *C. splendens* based on fluorescence microscopy (A). The occurrence of resilin is illustrated by blue colour according to its location in the wing (dorsal or/and ventral). White circles indicate firmly connected joints, lacking resilin. SEM images of the most distal part of the discoidal cell, the mediocubital bar (Ci,Di) and the arculus (Di,Ei). Dorsal and ventral side is indicated by downward pointing and upward pointing arrows, respectively. Slight differences between dorsal and ventral side are due to the use of different specimens. Ac - Arculus, CuA - cubital vein anterior, M - median vein, MA - median vein anterior, Mb - mediocubital bar, MP - median vein posterior, RA - radial vein anterior, RP - radial vein posterior. CLSM (maximum intensity projection), showing the occurrence of resilin in the arculus (Dii,Eii) and the mediocubital bar (Bii.Cii). Blue colour indicates the presence of resilin, red colours show strongly sclerotized cuticle, green colour show weakly sclerotized cuticle. Downward pointing and upward pointing arrows indicate the dorsal and ventral side of the wing, respectively. Slight differences between the location of the veins on the dorsal and ventral

side are due to the use of different specimens. Mechanical testing of wing deformability in *C. splendens*. Intact and cut forewings, were deflected from the dorsal and ventral side. Representative force displacement curve for a 10% deflection (F). Box-and-Whisker plots for resulting bending moments for the four experimental conditions (G). Resulting height profile before (H,I,L,M) and after (J,K,N,O) 10% deflection. Downward and upward pointing arrows indicate the dorsal (H-K) and the ventral (L-O) loadings, respectively. Corrugation profile of the *C. splendens* wing (P). Angles between neighbouring longitudinal veins are measured along five sections of the basal complex. Calculated angles are listed below each section. Cross sections through the basal complex of *C. splendens* (Q). Reconstructed micro-CT images show the corrugation of the wing in five section. Section 1 is most basal, section 5 is at the end of the basal complex. All sections are at equal distance to each other.

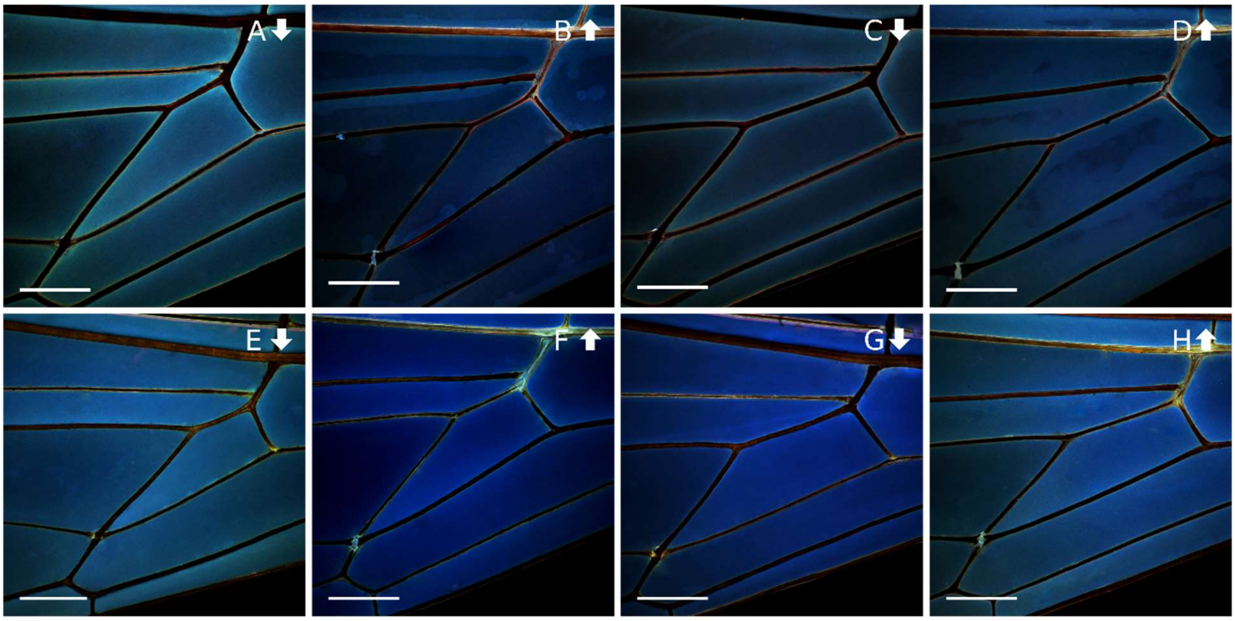


**Figure S6:** Confocal laser scanning micrographs (maximum intensity projection), showing the occurrence of resilin in the basal complex of *I. elegans*. Overlays of four different autofluorescence reveals the composition of the arculus and quadrilateral in female (A-D) and male (E-H), forewing (A,B,E,F) and hindwing (C,D,G,H) wings. Blue colour indicates the presence of resilin, red structures show relatively strongly sclerotised cuticle, green structures show weakly sclerotised cuticle. Downward pointing and upward pointing arrows indicate the dorsal and ventral side of the wing. Slight differences between dorsal and ventral side are due to the use of different specimen. The basal part of the wing is on the right, anterior part at the top. Scale bars = 300 µm.

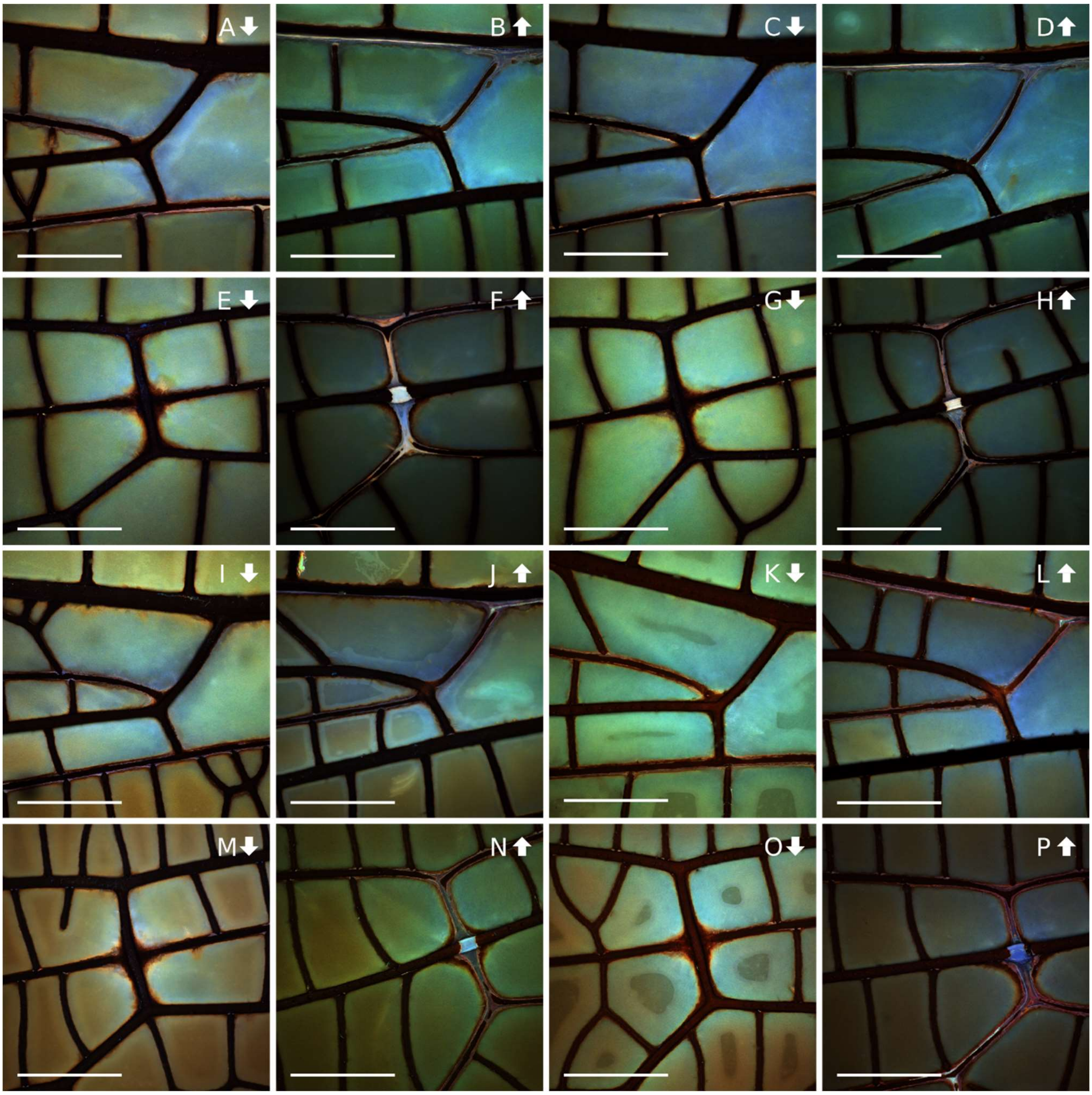


**Figure S7:** Confocal laser scanning micrographs (maximum intensity projection), showing the occurrence of resilin in the basal complex of *C. splendens*. Overlays of four different autofluorescence reveals the composition of the arculus (A-D, I-L) and the distal part of the quadrilateral (E-H, M-P) in female (A-H) and male (I-P), forewing (A,B,E,F,I,J,M,N) and hindwing (C,D,G,H,K,L,O,P) wings. Blue colour indicates the presence of resilin, red structures show relatively strongly sclerotised cuticle, green structures show weakly sclerotised cuticle. Downward pointing and upward pointing arrows indicate the dorsal and ventral side of the wing. Slight differences between dorsal and ventral side are due to the use of different specimen. The basal part of the wing is on the right, anterior part at the top. Scale bars = 500 µm.

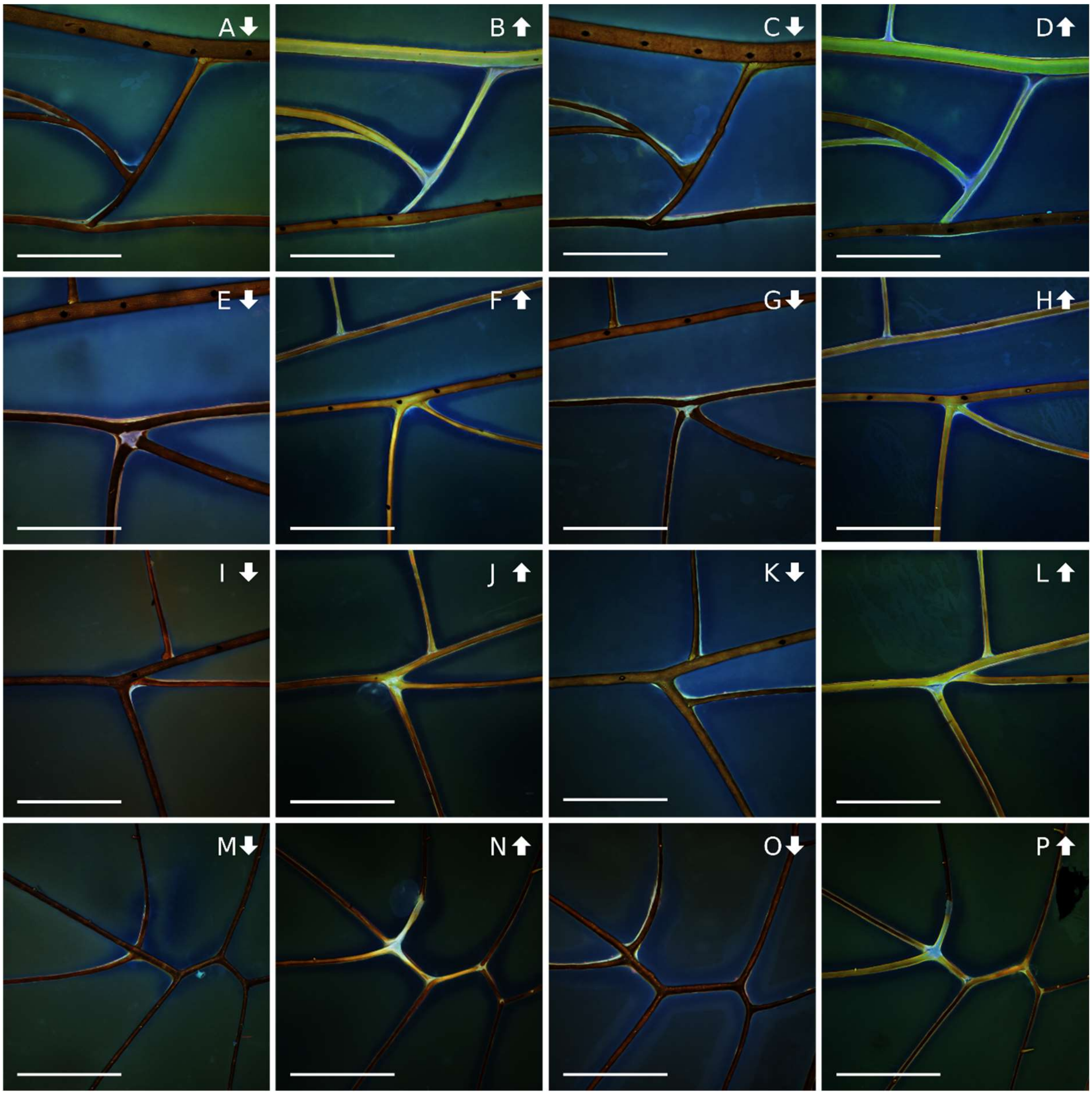


**Figure S8:** Confocal laser scanning micrographs (maximum intensity projection), showing the occurrence of resilin in the basal complex of the forewing of *S. vulgatum*. Overlays of four different autofluorescence reveals the composition of the arculus (A-D), the basal corner of the triangle (E-H), the apical corner of the triangle (I-L) and the posterior corner of the triangle (M-P) in female (A,B,E,F,I,J,M,N) and male (C,D,G,H,K,L,O,P) wings. Blue colour indicates the presence of resilin, red structures show relatively strongly sclerotised cuticle, green structures show weakly sclerotised cuticle. Downward pointing and upward pointing arrows indicate the dorsal and ventral side of the wing. Slight differences between dorsal and ventral side are due to the use of different specimen. The basal part of the wing is on the right, anterior part at the top. Scale bars = 500 µm.

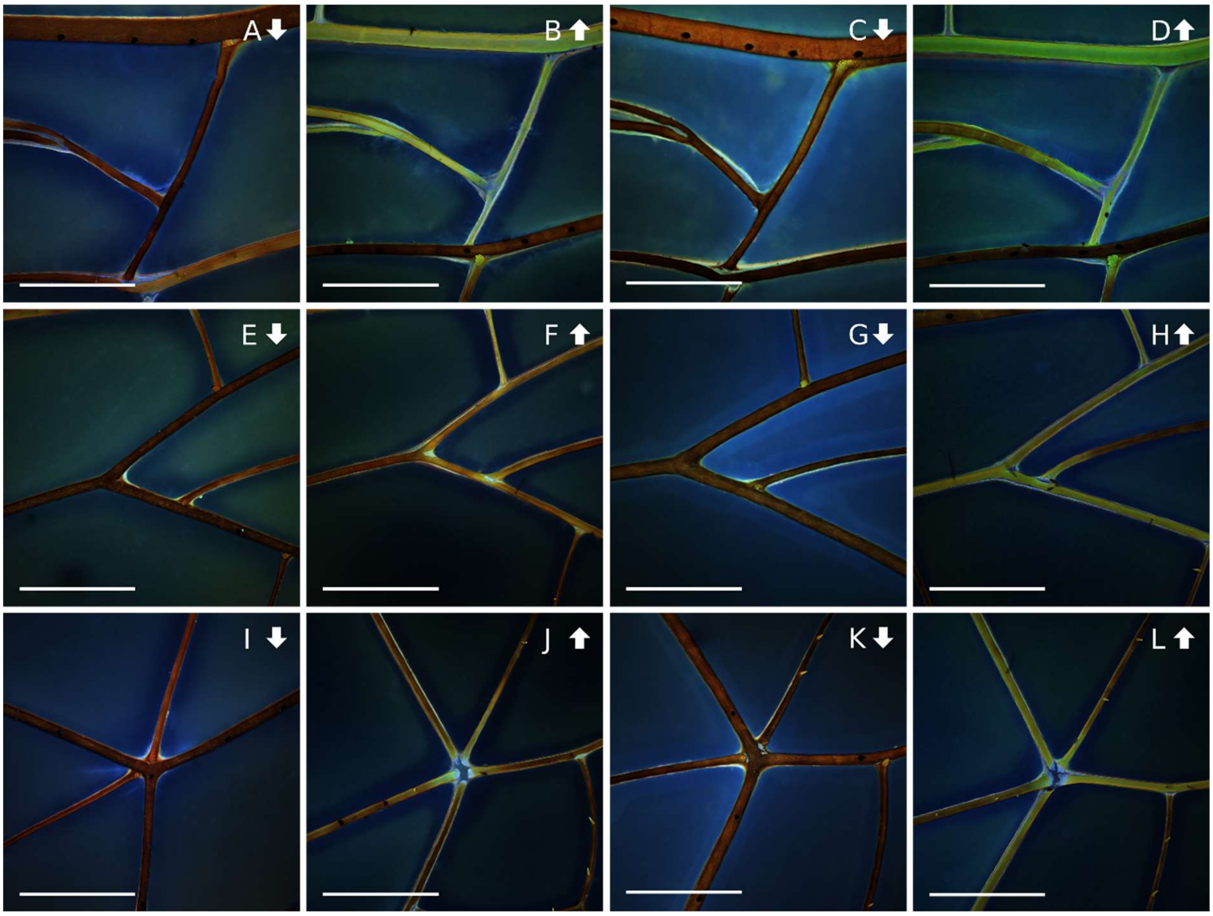


**Figure S9:** Confocal laser scanning micrographs (maximum intensity projection), showing the occurrence of resilin in the basal complex of the hindwing of *S. vulgatum*. Overlays of four different autofluorescence reveals the composition of the arculus (A-D), the apical corner of the triangle (E-H) and the posterior corner of the triangle (I-L) in female (A,B,E,F,I,J) and male (C,D,G,H,K,L) wings. Blue colour indicates the presence of resilin, red structures show relatively strongly sclerotised cuticle, green structures show weakly sclerotised cuticle. Downward pointing and upward pointing arrows indicate the dorsal and ventral side of the wing. Slight differences between dorsal and ventral side are due to the use of different specimen. The basal part of the wing is on the right, anterior part at the top. Scale bars = 500 µm.

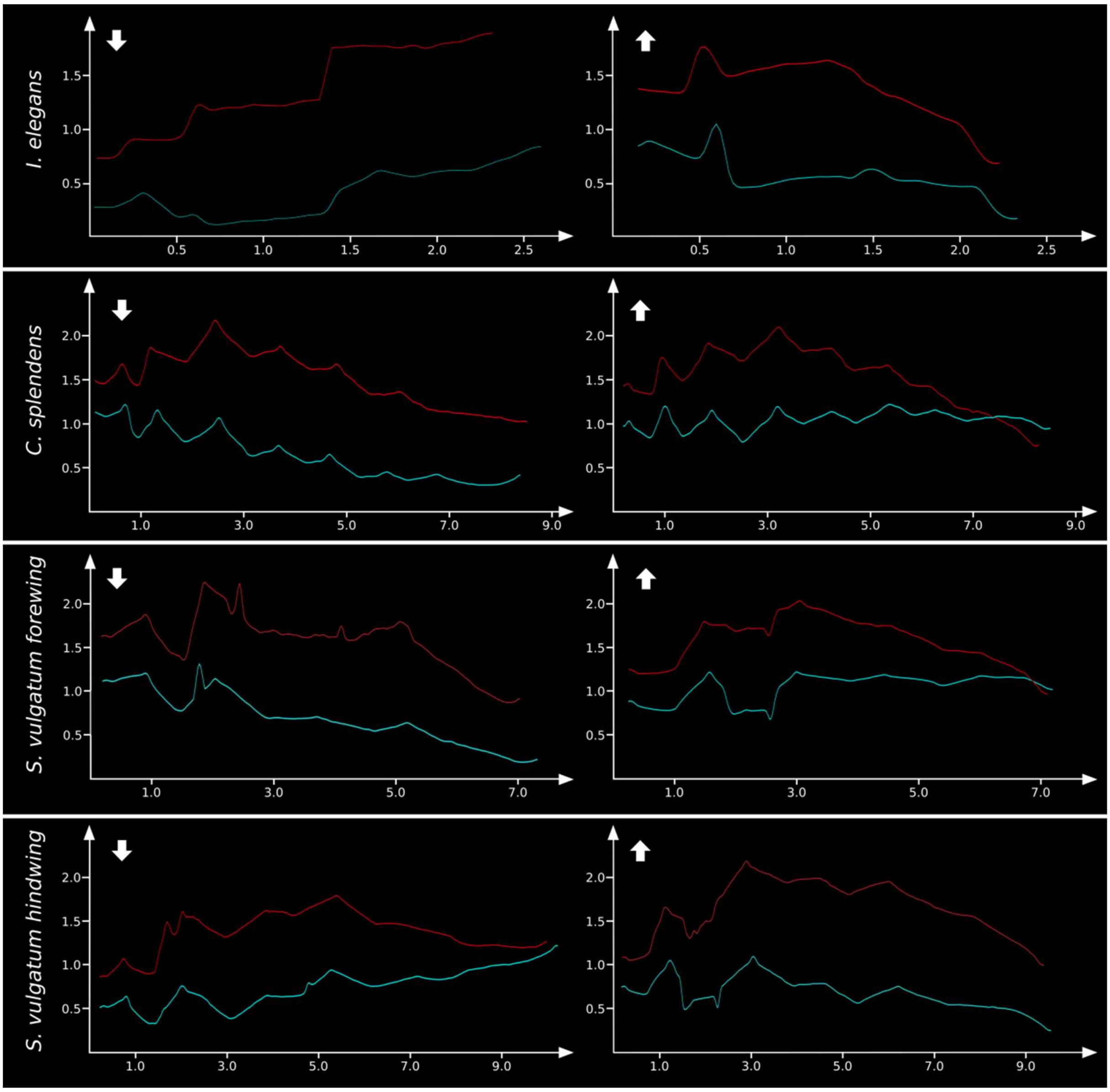


**Figure S10**: Representative height profiles of the edge of the basal complex in initial and deflected conditions (blue and red lines, respectively) in *I. elegans*, *C. splendens* and *S. vulgatum*. Downward and upward pointing arrows indicate the dorsal and ventral loadings, respectively.

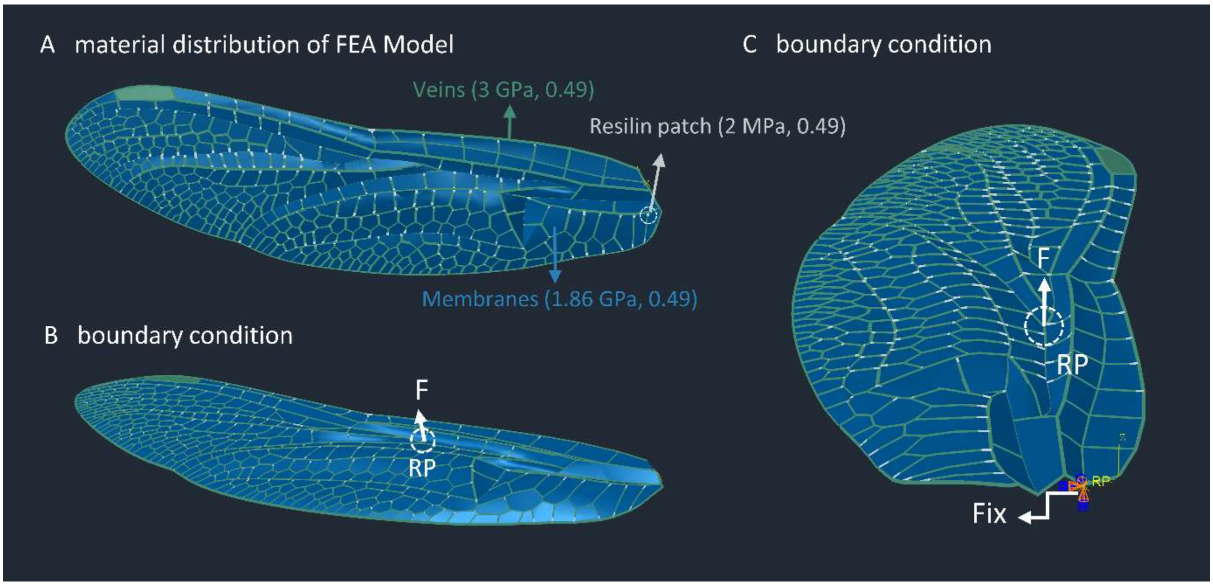


**Figure S11.** Material distribution and boundary condition of FEA model of the entire wing of dragonfly *S. vulgatum*.

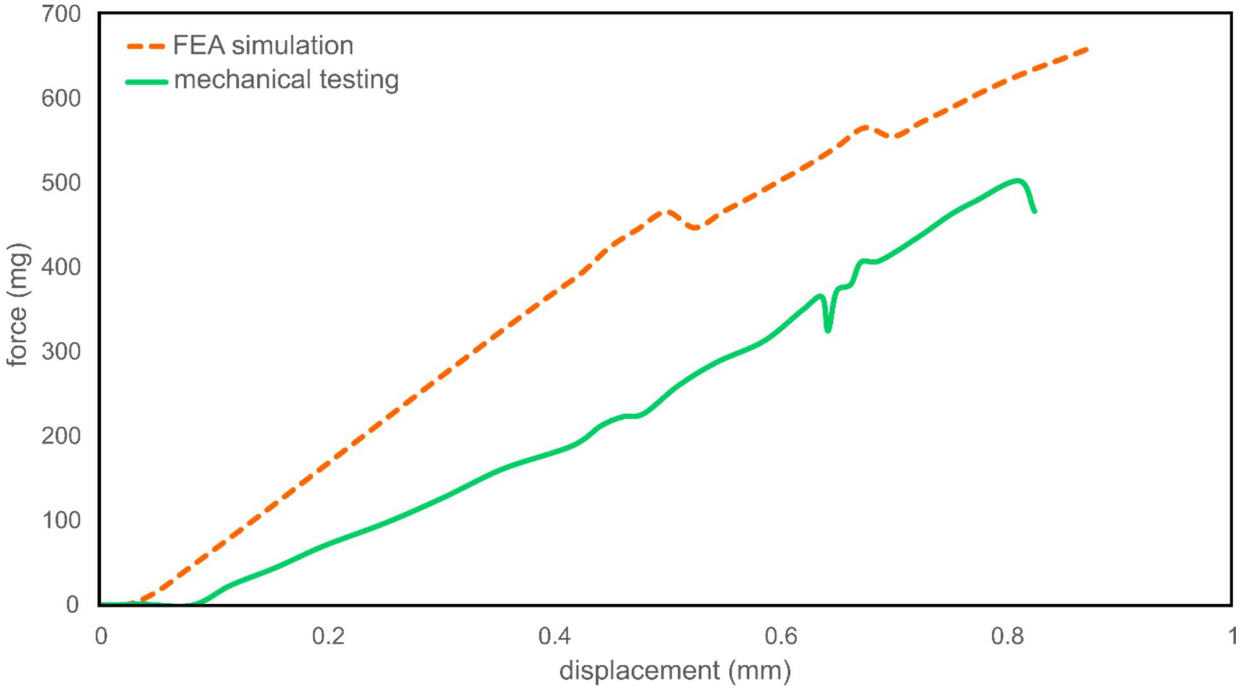


**Figure S12.** Validation of the FEA simulation of the forewing of *S. vulgatum*.

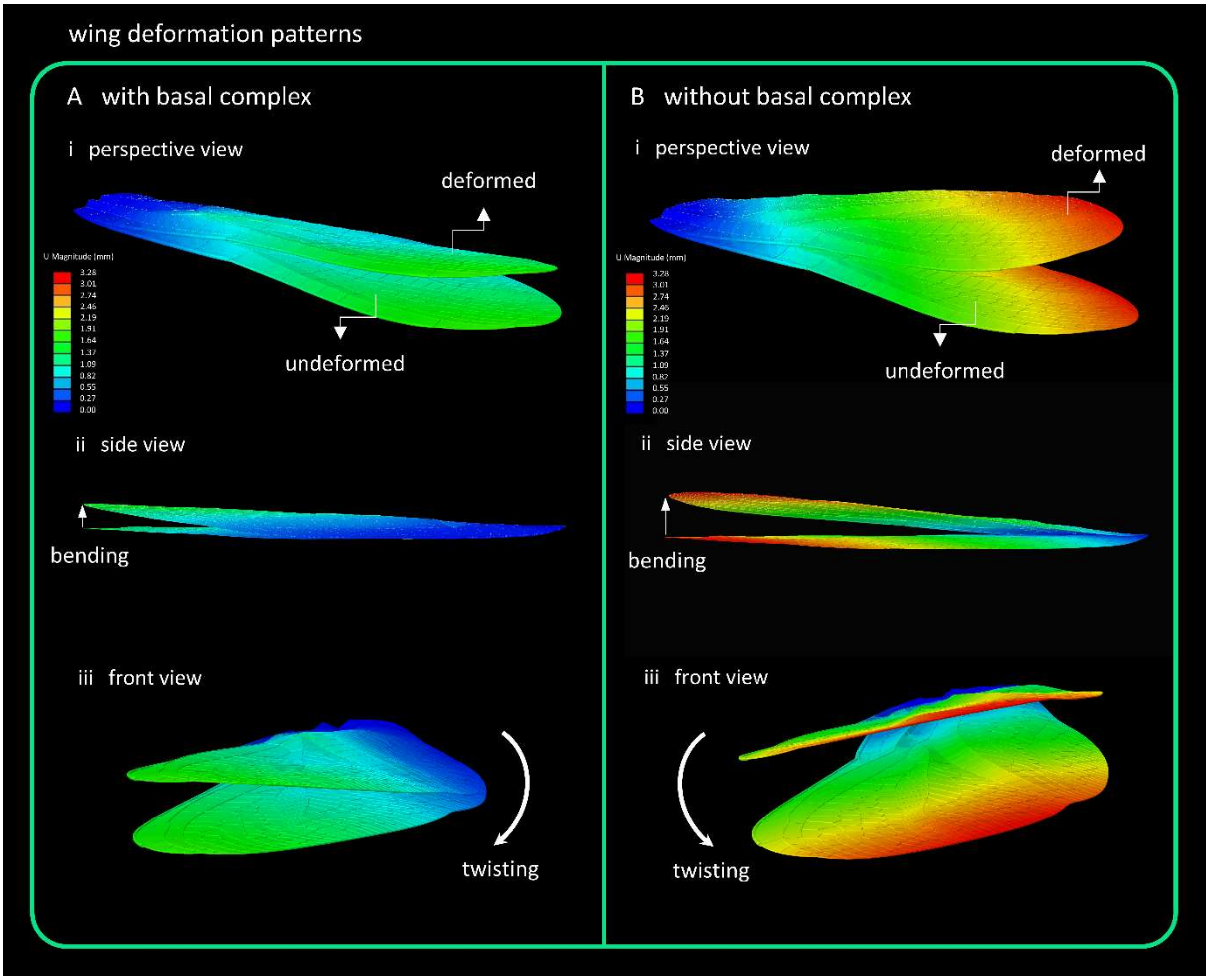


**Figure S13**. Finite element analysis and deformation pattern of the entire wing of dragonfly *S. vulgatum* with basal complex (A) and without basal complex (B). Both models are shown in deformed and undeformed states from perspective view (Ai, Bi), side view (Aii, Bii) and front view (Aiii, Biii).

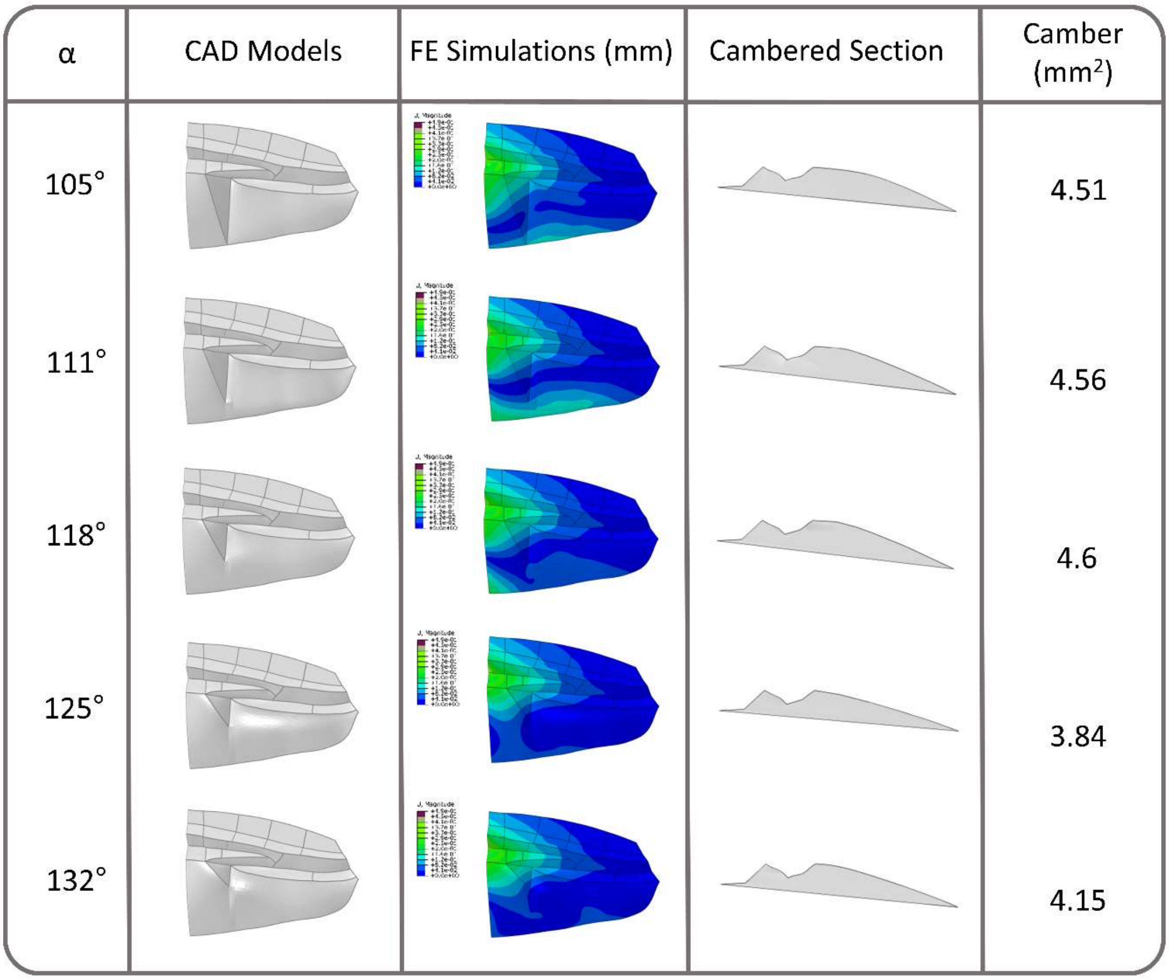


**Figure S14**. Finite element analysis of the effect of triangle exterior angle (α) variations on camber formation capability of the basal complex of dragonfly *S. vulgatum*.

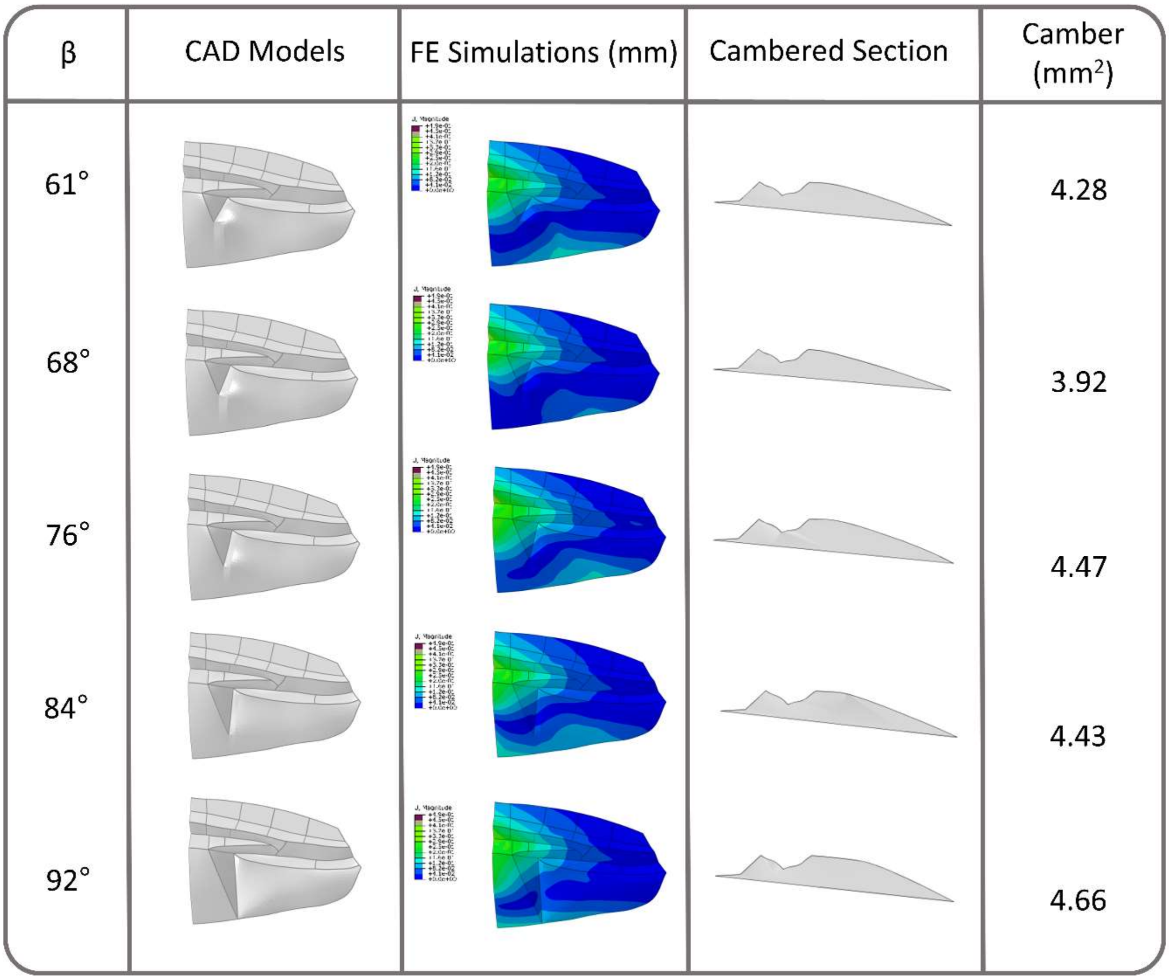


**Figure S15**. Finite element analysis of the effect of triangle interior angle (β) variations on camber formation capability of the basal complex of dragonfly *S. vulgatum*.

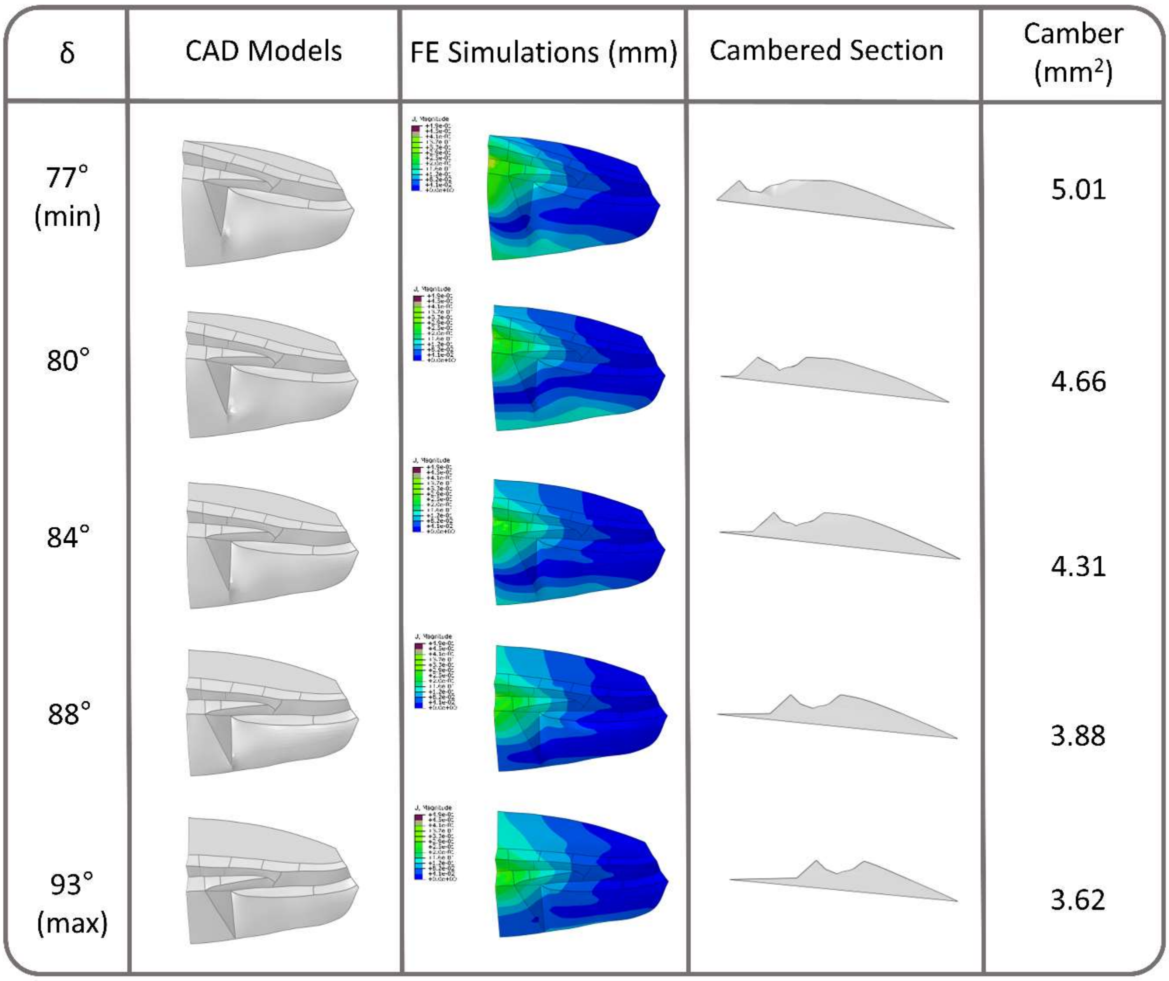


**Figure S16.** Finite element analysis of the effect of longitudinal veins rotation angle (δ) variations on camber formation capability of the basal complex of dragonfly *S. vulgatum*.

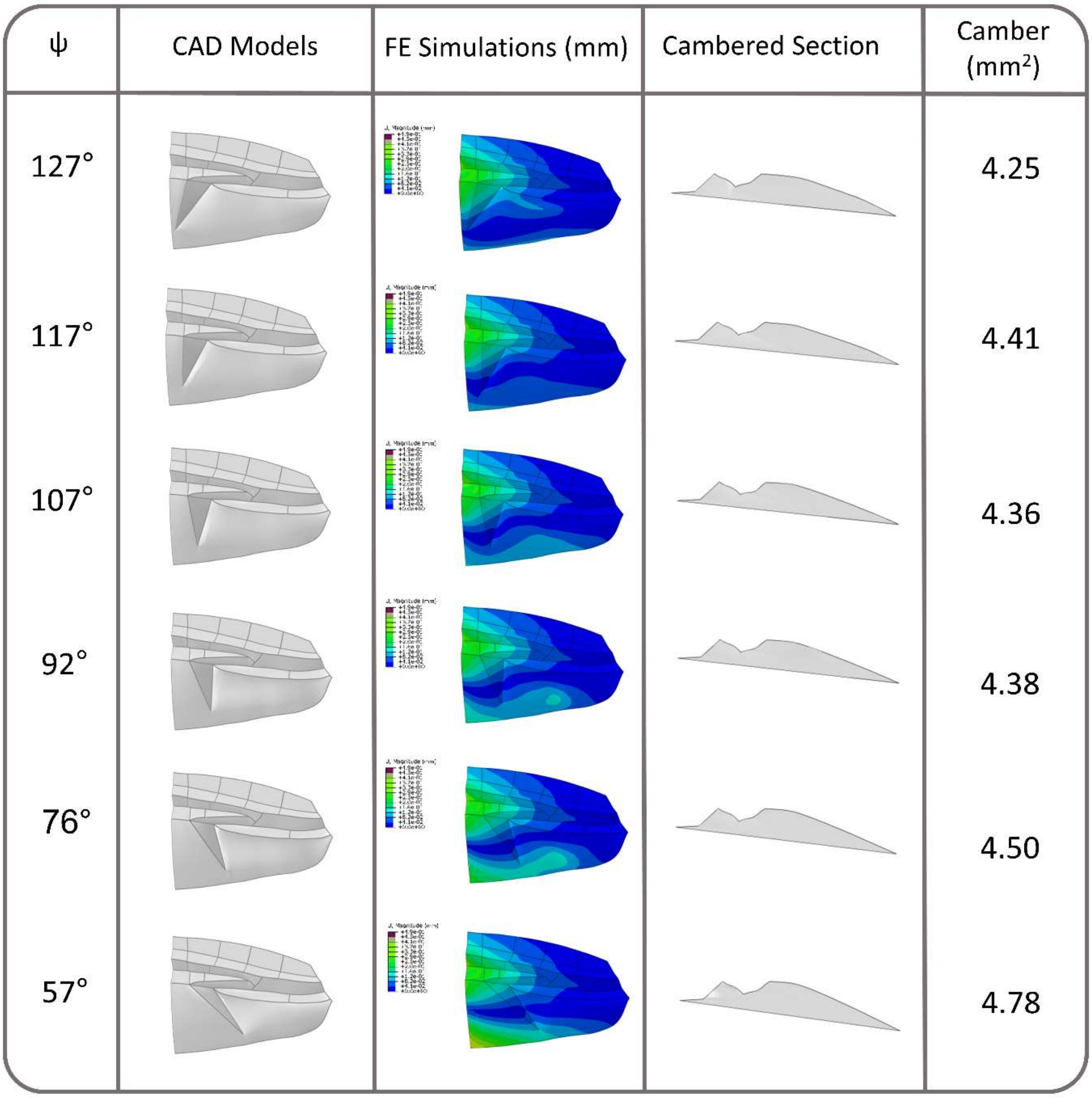


**Figure S17**. Finite element analysis of the effect of triangle rotation angle (ψ) variations on camber formation capability of the basal complex of dragonfly *S. vulgatum*.

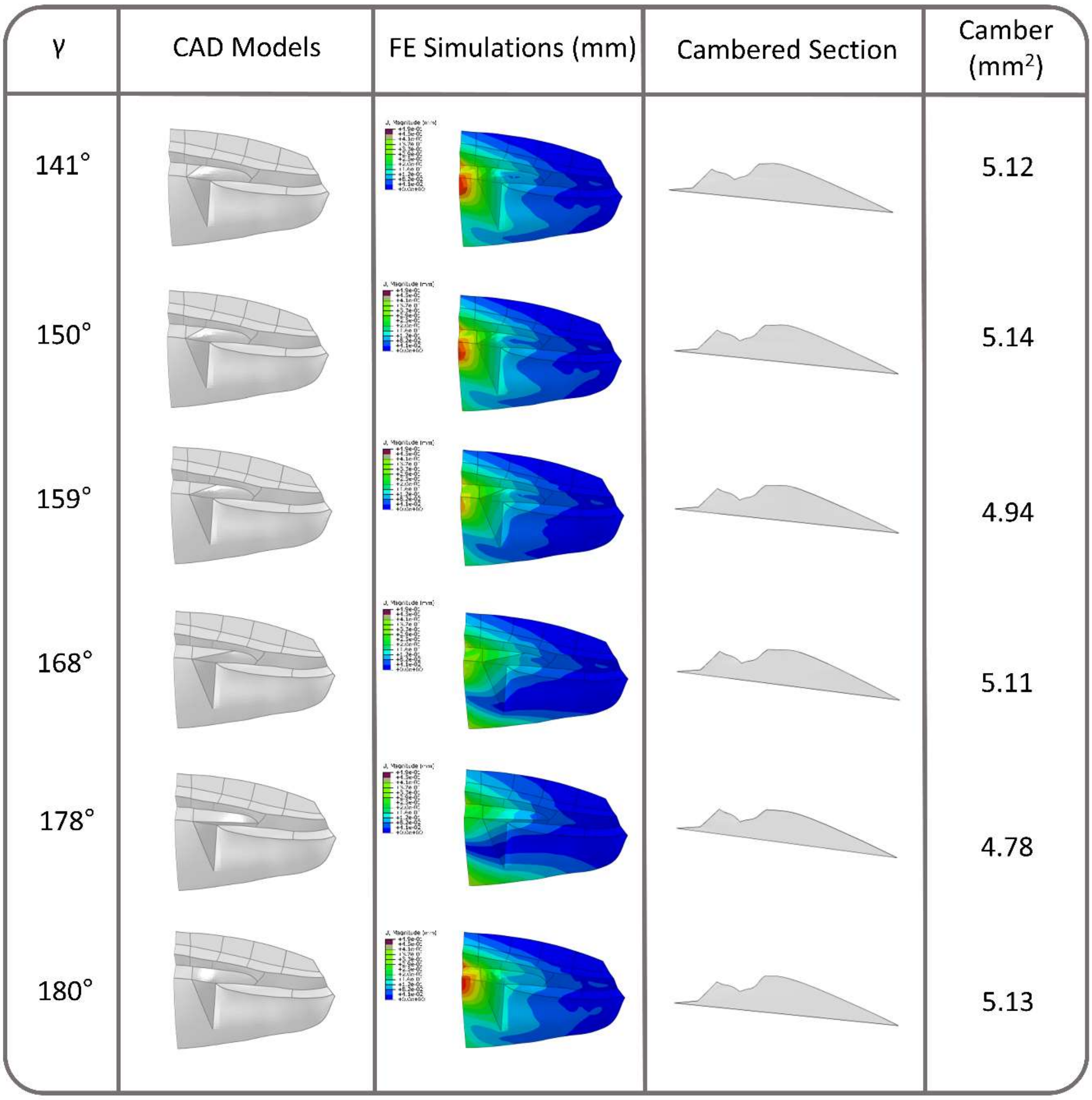


**Figure S18.** Finite element analysis of the effect of subtriangle rotation angle (γ) variations on camber formation capability of the basal complex of dragonfly *S. vulgatum.*

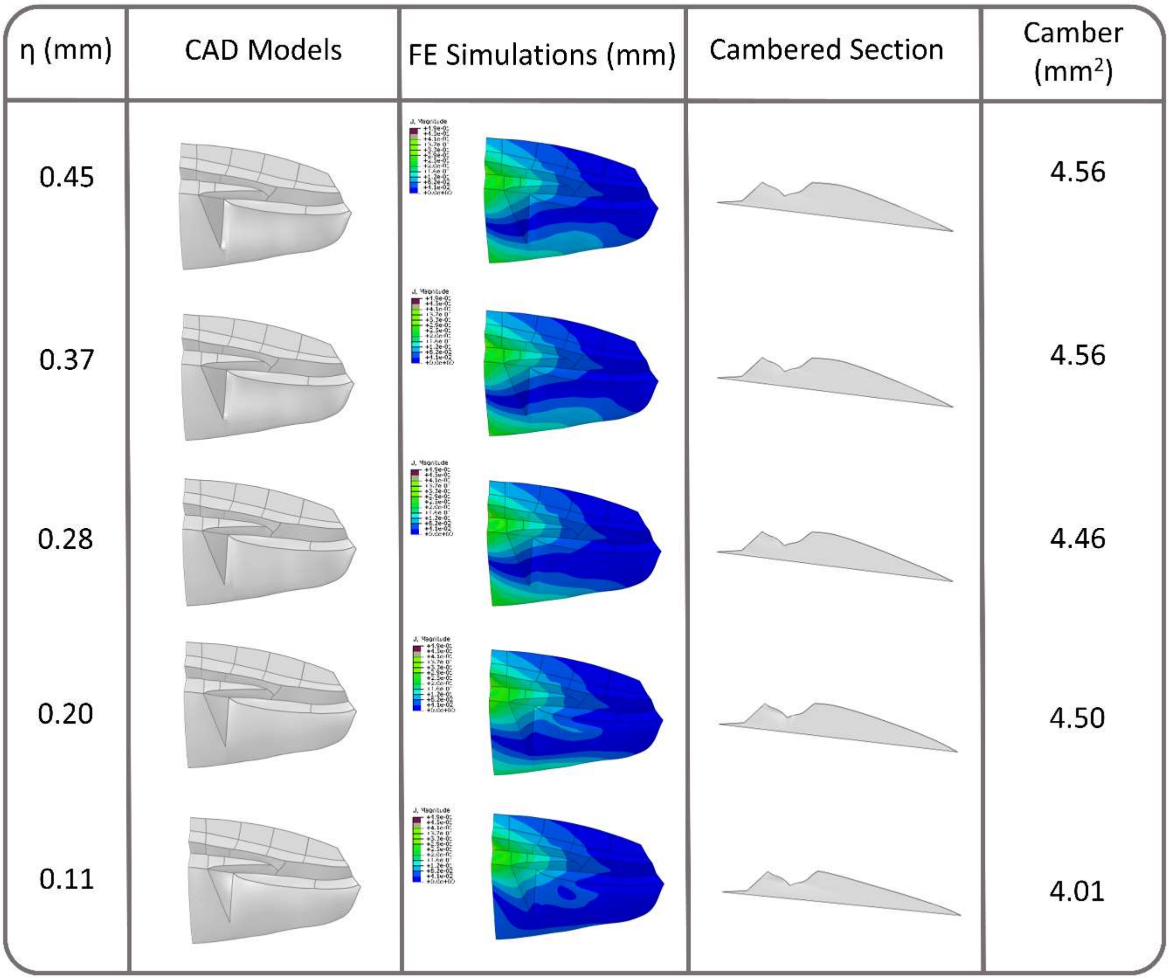


**Figure S19**. Finite element analysis of the effect of triangle incline depth (η) variations on camber formation capability of the basal complex of dragonfly *S. vulgatum*.

Camber area calculation

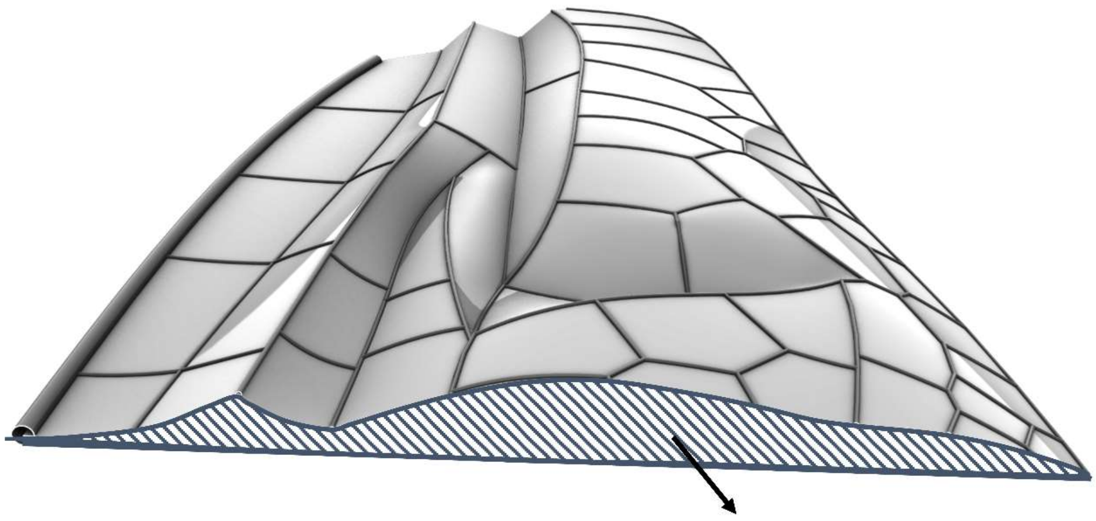


**Figure S20.** Camber area calculation of the basal complex of dragonfly *S. vulgatum.*

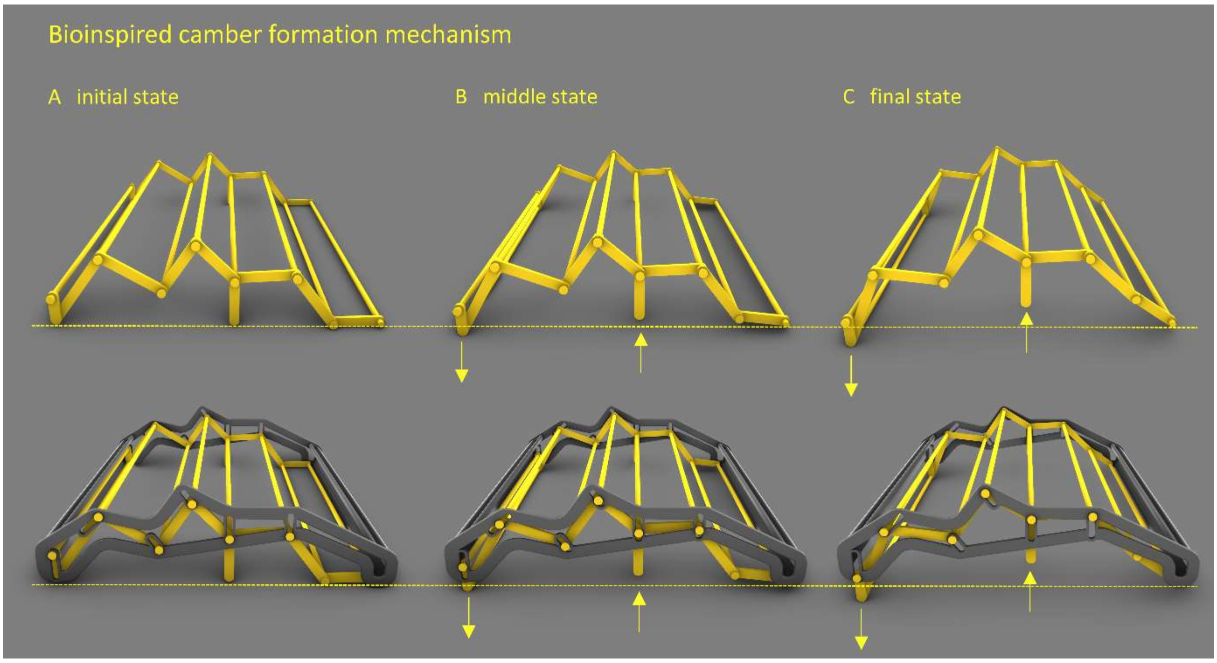


**Figure S21.** Bioinspired camber formation mechanism in (A) initial state, (B) middle state, and (C) final state.

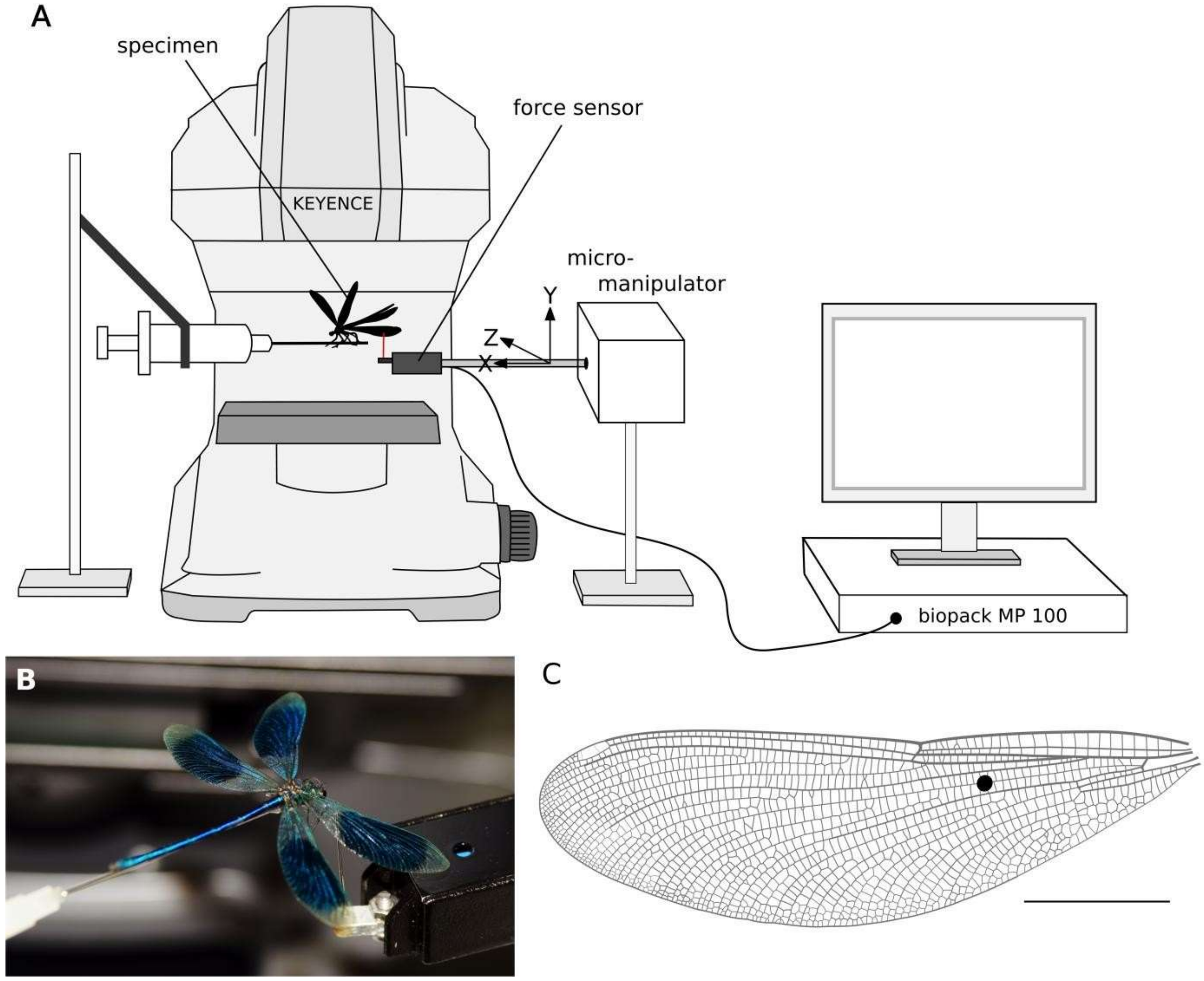


**Figure S22:** Experimental setup for measuring the deformability of Odonata wings. (A) Schematic diagram of the important elements used to generate forces as well as height profiles of the wing during deflection. The wing of the specimen is positioned above the needle attached to the force sensor (illustrated in red). The force is applied at the radial vein posterior (RP), about 30% of the entire length of the wing way from the fixed wing base (B). A black circle indicates the exact location of the applied force on a *C. splendens* wing (C). Scale bar: 7.5 mm.

## Other supplementary materials

**Video S1.** 3D reconstruction of the basal complex of the forewing of the dragonfly *Sympetrum vulgatum*.

**Video S2.** 3D reconstruction of the basal complex of the hindwing of the dragonfly *Sympetrum vulgatum*.

**Video S3.** 3D reconstruction of the basal complex of the forewing of the dragonfly *Ischnura elegans*.

**Video S4.** 3D reconstruction of the basal complex of the forewing of the dragonfly *Calopteryx splendens*.

**Video S5.** Bioinspired mechanism: 3d rendered model and 3d printed part.

**Table S1:** Adjustments for the micro-CT

| Species | Wing | Voltage [kV] | Current [mA] | Pixel size [µm] |
|---|---|---|---|---|
| *Ischnura elegans* | forewing | 37 | 234 | 2.67 |
| *Calopteryx splendens* | forewing | 35 | 216 | 3.60 |
| *Sympetrum vulgatum* | forewing | 40 | 250 | 3.40 |
| | hindwing | 40 | 250 | 5.00 |

**Table S2:** Forces and bending moments during mechanical testing for each experimental trail.

| Species | Specimen | Wing | Force [mg] | | | | Bending moment [mg*mm] | | | |
|---|---|---|---|---|---|---|---|---|---|---|
| | | | dorsal | dorsal half | ventral | Ventral half | dorsal | Dorsal half | Ventral | Ventra. half |
| *I. elegans* | 1 | Forewing | 58.470 | 32.247 | 94.304 | 85.626 | 28.216 | 14.199 | 43.077 | 39.763 |
| | 2 | Forewing | 62.350 | 94.032 | 75.367 | 78.102 | 25.464 | 39.719 | 31.776 | 32.890 |
| | 3 | Forewing | 36.939 | 35.547 | 30.714 | 19.143 | 15.078 | 15.990 | 13.122 | 8.744 |
| | 4 | Forewing | 51.665 | 44.876 | 53.761 | 46.756 | 22.506 | 18.149 | 22.900 | 19.230 |
| | 5 | Forewing | 38.111 | 30.544 | 78.687 | 102.413 | 16.739 | 13.100 | 31.619 | 44.866 |
| *C. splendens* | 1 | Forewing | 208.882 | 173.251 | 173.313 | 208.859 | 132.582 | 120.151 | 123.311 | 151.642 |
| | 2 | Forewing | 153.747 | 104.804 | 99.789 | 74.905 | 114.780 | 78.788 | 75.034 | 56.850 |
| | 3 | Forewing | 181.588 | 143.604 | 159.571 | 142.434 | 140.230 | 99.859 | 114.078 | 104.383 |
| | 4 | Forewing | 149.310 | 122.689 | 240.059 | 71.670 | 108.978 | 87.548 | 174.652 | 54.221 |
| | 5 | Forewing | 259.874 | 153.698 | 275.419 | 274.193 | 181.892 | 113.504 | 194.287 | 202.857 |
| *S. vulgatum* | 1 | Forewing | 317.636 | 191.346 | 221.128 | 187.243 | 231.663 | 142.191 | 163.983 | 136.299 |
| | 1 | Hindwing | 150.211 | 91.206 | 97.071 | 91.622 | 104.201 | 65.921 | 64.508 | 62.823 |
| | 2 | Forewing | 646.131 | 534.308 | 377.716 | 210.072 | 394.783 | 319.850 | 225.506 | 126.295 |
| | 2 | Hindwing | 273.322 | 110.543 | 263.687 | 64.393 | 169.267 | 70.453 | 168.689 | 42.142 |
| | 3 | Forewing | 185.153 | 202.916 | 251.892 | 240.613 | 140.906 | 151.770 | 189.100 | 180.369 |
| | 3 | Hindwing | 114.971 | 113.824 | 124.155 | 88.767 | 79.545 | 80.032 | 85.861 | 62.068 |
| | 4 | Forewing | 130.649 | 112.859 | 111.208 | 109.492 | 98.457 | 85.050 | 83.895 | 82.513 |
| | 4 | Hindwing | 97.679 | 97.508 | 151.272 | 151.272 | 64.093 | 63.981 | 99.258 | 99.258 |
| | 5 | Forewing | 255.121 | 240.194 | 245.263 | 156.978 | 186.932 | 175.995 | 179.709 | 115.021 |
| | 5 | Hindwing | 378.802 | 227.089 | 271.500 | 216.203 | 251.737 | 150.915 | 180.428 | 143.680 |

## Supplementary References

[1] Gorb, S. N. Serial elastic elements in the damselfly wing: mobile vein joints contain resilin. *Naturwissenschaften* **86** 552–555 (1999).

[2] Donoughe, S., Crall, J. D., Merz, R. A., Combes, S. A. Resilin in dragonfly and damselfly wings and its implications for wing flexibility. *J Morphol* **272** 1409–1421 (2011).

[3] Appel, E., Gorb, S. N. Resilin-bearing wing vein joints in the dragonfly *Epiophlebia superstes*. *Bioinspir Biomim* **6** 046006 (2011).
