# Supplementary material for: Basal complex: a smart wing component for automatic shape morphing
Source: Commun Biol. 2023 Aug 17;6:853. doi: 10.1038/s42003-023-05206-1 (PMC10435446; doi:10.1038/s42003-023-05206-1)
Supplement: Supplementary file 3 — Description of Additional Supplementary Files [file 42003_2023_5206_MOESM3_ESM.pdf]

Description of Additional Supplementary Files

File name: Video S1
Description: 3D reconstruction of the basal complex of the forewing of the dragonfly Sympetrum vulgatum.

File name: Video S2
Description: 3D reconstruction of the basal complex of the hindwing of the dragonfly Sympetrum vulgatum.

File name: Video S3
Description: 3D reconstruction of the basal complex of the forewing of the dragonfly Ischnura elegans.

File name: Video S4
Description: 3D reconstruction of the basal complex of the forewing of the dragonfly Calopteryx splendens.

File name: Video S5
Description: Bioinspired mechanism: 3d rendered model and 3d printed part.
